# The prognostic role of microRNA in epithelial ovarian cancer: a systematic review of literature with an overall survival meta-analysis

**DOI:** 10.18632/oncotarget.27246

**Published:** 2020-03-24

**Authors:** Patricia Ferreira, Rosimeire Aparecida Roela, Rossana Veronica Mendoza Lopez, Maria Del Pilar Estevez-Diz

**Affiliations:** ^1^ Instituto do Cancer do Estado de Sao Paulo, Faculdade de Medicina da Universidade de Sao Paulo, Sao Paulo, Brazil

**Keywords:** ovarian cancer, microRNA, miRNA, prognostic factors, overall survival

## Abstract

**Objective:** To accomplish a systematic review of literature with overall survival meta-analysis about the role of microRNA in epithelial ovarian cancer as prognostic and predictive factor to chemotherapy response.

**Methods:** A search was conducted in the PubMed database, using the keywords “microRNA” and “ovarian cancer” or “miRNA” and “ovarian cancer”. Original articles published before 02/02/2019 that had as main subject microRNA (miRNA) and ovarian cancer were included. We considered for inclusion only studies that associated microRNA to chemotherapy-related diagnosis, prognosis, or response in ovarian cancer.

**Results:** The literature search returned 1,482 articles, 497 of which fulfilled inclusion criteria, yielding 350 miRNAs. The status of each miRNA was assessed in serum and tissue of ovarian cancer, benign tumors, and healthy tissue. The status of up-/downregulation of miRNAs was related to prognostic features (overall survival and disease-free survival) and response predictive features such as platinum and paclitaxel sensitivity/resistance. The miRNAs that had been cited three or more times were selected for prognostic and response predictive features analysis. Twelve miRNAs fulfilled all these criteria and were included in the overall survival meta-analysis.

**Conclusions:** miRNAs affect virtually all mechanisms of carcinogenesis, working as either oncogenes or tumor suppressor genes. In this systematic review we identified miRNAs that may be related to prognosis, diagnosis, and chemotherapy sensitivity. The 12 miRNAs identified here should be included in future studies for validation.

## INTRODUCTION

**Figure 1:**
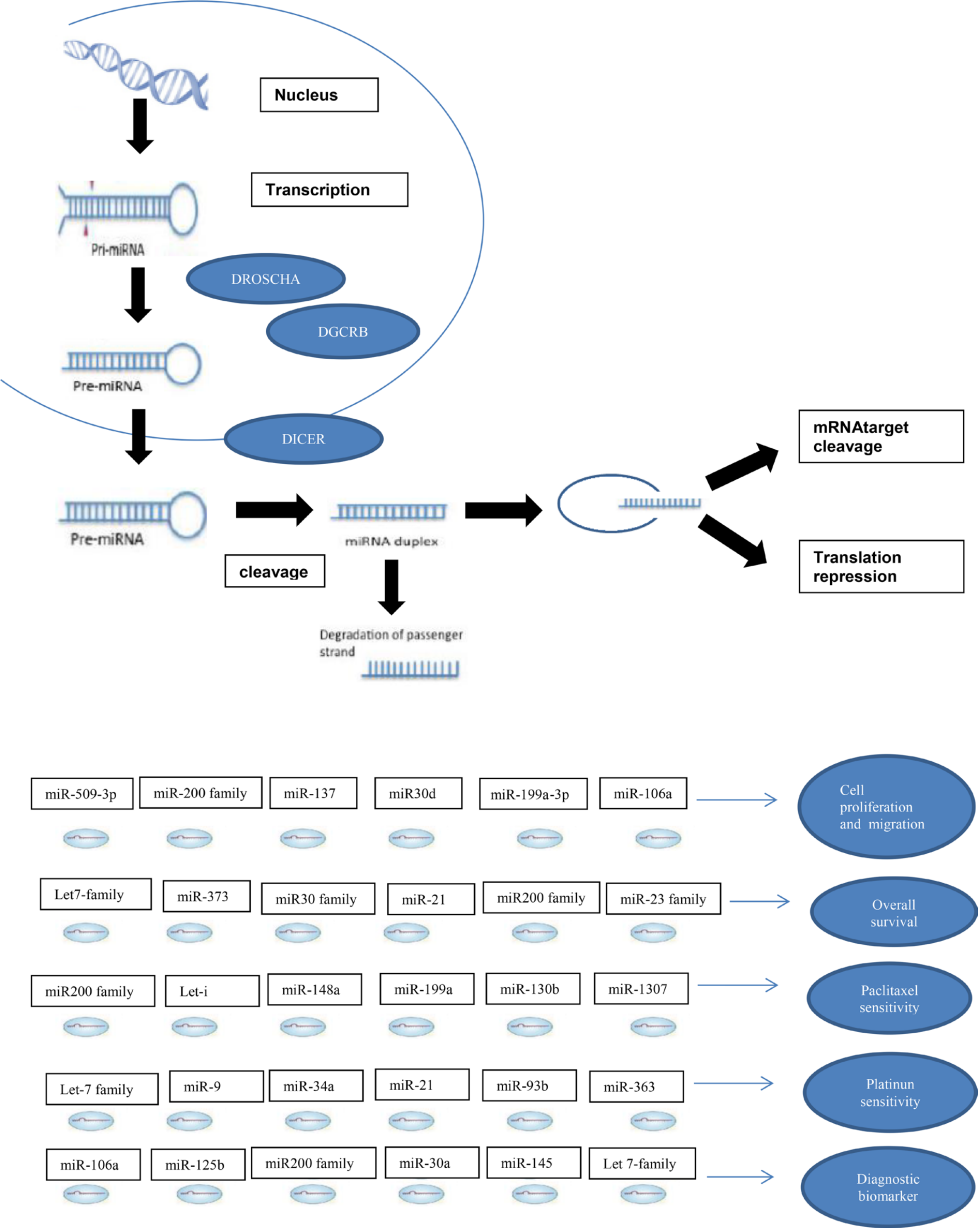
miRNA in ovarian cancer.

Ovarian cancer is the eighth most common female cancer worldwide and the eighth in mortality [1]. The 5-year survival rate for patients with advanced ovarian cancer is 30%, despite the use of platinum-based chemotherapy and taxanes [2]. The high mortality is associated with difficulties in early detection because ovarian cancer rarely causes subjective symptoms; 40% to 50% of patients have the diagnosis in advanced stages (III or IV) [3], and safe, minimally invasive procedures for early detection have not been established. Improvements in ovarian cancer diagnosis and more precise instruments for prognostic determination may improve the odds of survival [3]. microRNA (miRNA) are small, non-coding RNA molecules of 20-22 base pairs length that function in transcriptional and post-transcriptional regulation of gene expression by targeting complementary sequences of DNA and mRNA [4]. miRNAs have been shown to affect virtually all cellular functions, including proliferation, apoptosis, cell cycle, and differentiation, and have been associated with several cancers. In epithelial ovarian cancer, several miRNAs are abnormally expressed and may act as either tumor suppressors or oncogenes (Figure 1) [4].

## MATERIALS AND METHODS

### Search strategy

A PubMed search was conducted by two independent researchers using the terms “microRNA” and “ovarian cancer” or “miRNA” and “ovarian cancer”, and with a publication date prior to 2/2/2019. Each unique article was then evaluated according to the following criteria:

### Inclusion criteria

-Studies that demonstrated a relationship between miRNA expression levels in epithelial ovarian cancer and:

-Overall survival (OS)

-Disease free survival

-Resistance to chemotherapy (cisplatin and/or paclitaxel)

-Sensitivity to chemotherapy (cisplatin and/or paclitaxel)

-Cell proliferation and/or migration

### Exclusion criteria

-Studies that addressed tumors other than ovarian cancer

-Studies that addressed non-epithelial ovarian cancer

-Studies that do not address ovarian cancer

-Review articles

-Studies in animal tissue

The articles were evaluated for inclusion by two independent researchers and discrepancies were resolved by a third researcher. The PRISMA checklist was followed for systematic review building. http://prisma-statement.org/PRISMAStatement/PRISMAStatement.aspx.

Most of the miRNAs found had been cited only in one or two articles and we considered this insufficient evidence for analysis. Therefore, only miRNAs cited in three or more papers were considered for final analysis.

### Statistical methods

For construction of the meta-analysis correlating miRNA expression level to OS we used the hazard ratio (HR) as the association measure and a confidence interval of 95%. A forest plot graphic was used to visualize the results. Heterogeneity of studies was evaluated using the *I*^2^ test under null hypothesis of no heterogeneity between studies. The resume measures were calculated for family whereas the value of sample size as weight of each study. The forest plot graphic represents the measure of association of each study. All analyses were calculated in STATA software for Windows v1.1.

## RESULTS

### Systematic review

**Figure 2:**
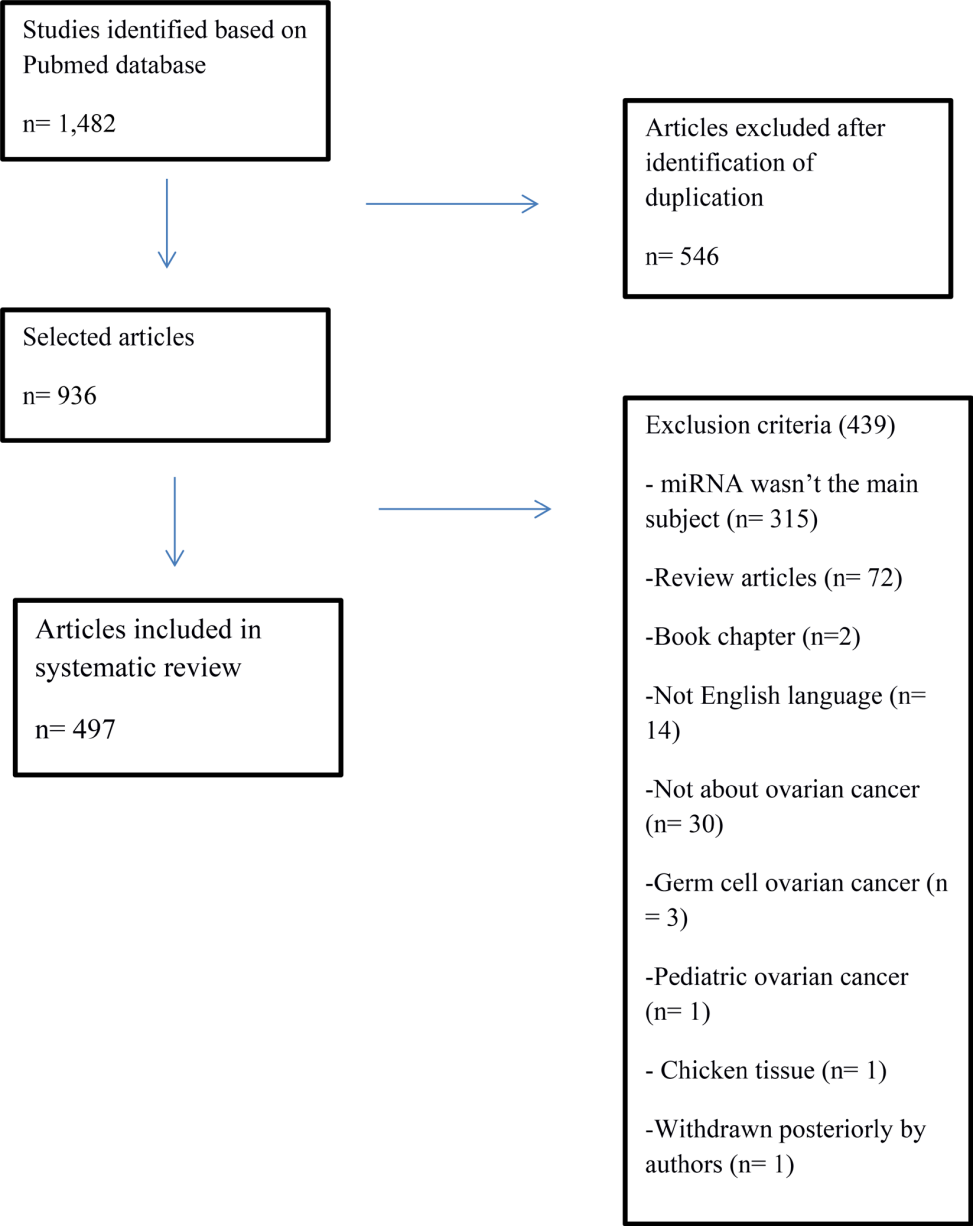
Flowchart of identification and selection of articles to literature systematic review.

**Figure 3:**
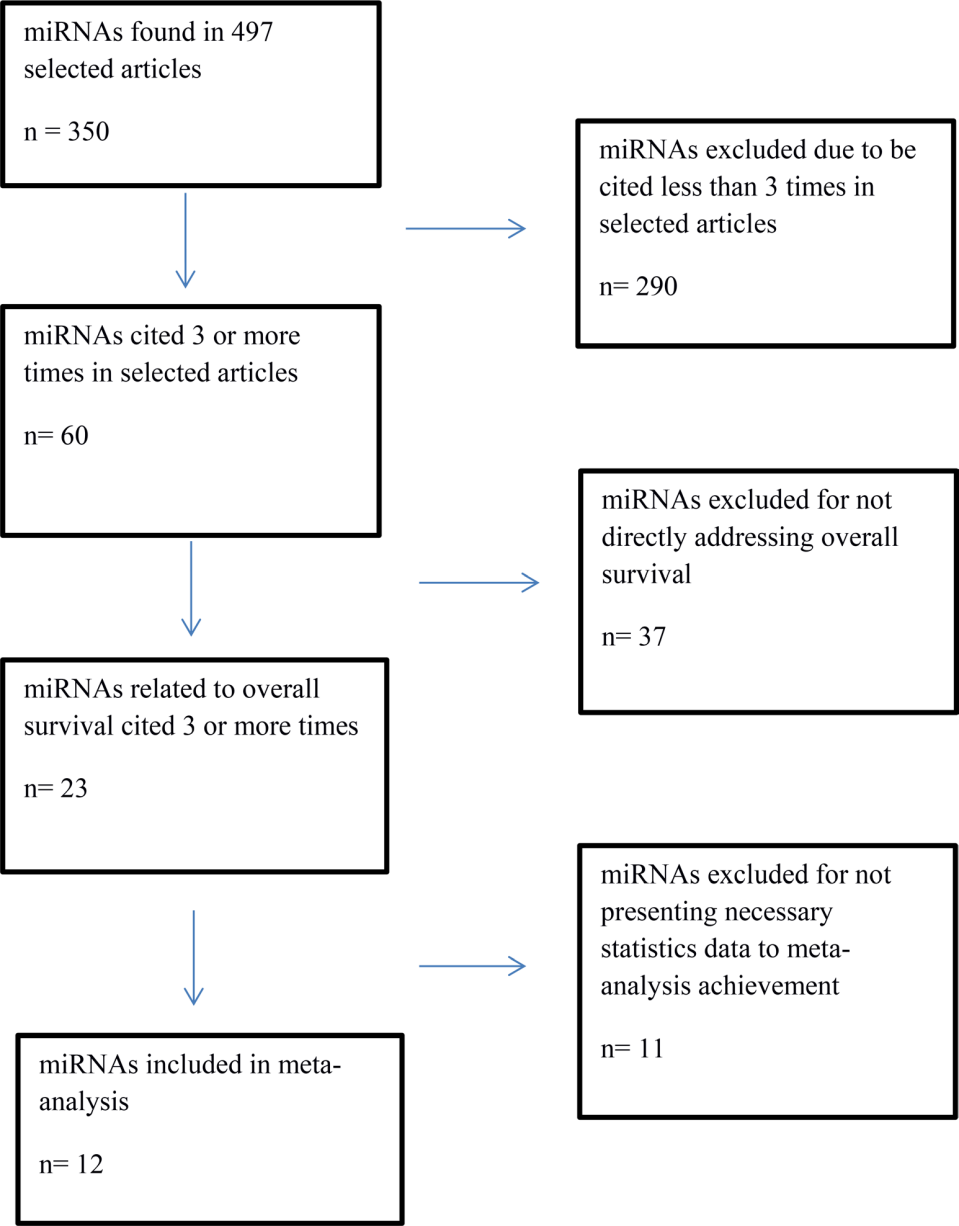
Flowchart of selection of miRNAs to overall survival meta-analysis.

Using the keywords “ovarian cancer” and “miRNA” or “ovarian cancer” and “microRNA”, 1,482 articles were identified. After the exclusion of 546 duplicate articles, 936 were evaluated according to inclusion and exclusion criteria. Finally, 497 articles were selected for systematic review (Figure 2). In these eligible articles were described 350 miRNAs (Figure 3).

The majority of the miRNAs identified were cited one or two times in the 497 articles selected. With the intention to select the ones more cited in the literature and to study these miRNA with more complexity, we reviewed only the miRNA that had been cited three or more times.

First, expression levels of miRNAs in ovarian cancer samples were compared to those of healthy/benign samples. We found in the articles included in this study that ovarian cancer samples showed higher levels of miR-125b, miR-126, miR-1307, miR-182, miR-23a, miR-27a, miR-200b, miR-200c-3p, miR-205, miR-221, miR-30d, miR-31, miR-375, miR-429, miR-509-3p, miR-9, let-7a, let-7d, let-7c, and let-7f. In contrast, ovarian cancer samples showed lower levels of miR-139-5p, miR-149, miR-199a-3p, miR23b, miR363, miR-409-3p, and miR-494 (Supplementary Table 1).

The miR-106a, miR-145, miR-148a, miR-152, miR-199a, miR-497, miR-93, and let-7 family presented more than one discrepancy in their expression among ovarian cancer samples compared to healthy/benign sample. We identified that miR-155, miR-200a, miR-200c, miR-506, miR-130b, miR-133a, miR-137, miR-141, miR-193b, miR-21, miR-22, miR-25, miR-29b, miR-335 and let-7b were upregulated in ovarian cancer samples compared to healthy/benign sample in all articles included in this systematic review, except one in each group which was downregulated**.** (Supplementary Table 1). miRNAs related to cell proliferation were also evaluated and upregulation of miR-106a, miR-182, miR-25, miR-200a, miR-200c-3p, miR-203, miR-205, miR-221, miR-30d, and miR-603 was found to be related to increased cell proliferation. In contrast, miR-125b, miR-130b, miR-133a, miR-137, miR-145, miR-148a, miR-149, miR-152, miR-199a, miR-22, miR-23b, miR-29b, miR-200c, miR215, miR-34a, miR-34c, miR-363, miR-429, miR-494, miR-497, miR-506, miR-509-3p, miR-9, and let-7b reduces cell proliferation when upregulated. Downregulation of miR-193b and miR-199a-3p is related to increased cell proliferation (Supplementary Table 2).

miRNAs related to OS were analyzed and decreased OS was associated with the downregulation of miR-106a, miR-139, miR-145, miR-148a, miR-182, miR-22, miR-23b, miR-29b, miR-30a, miR-335, and miR-497, and with upregulation of miR-141, miR-149, miR-20a, miR-21 miR-23a, miR-25, miR-27a, miR-200b, miR-203, miR-221, miR-30d, and miR-363 in articles included in this study. There was a relationship between increased OS and upregulation of miR-506 and miR-9 and with downregulation of let-7a and let-7d in these articles. We identified a discrepancy between upregulated and downregulated status of miR-200a, miR200c, miR-200c-3p, miR-30d, miR-429, and miR-509-3p and the length of overall survival (Supplementary Table 3).

In terms of resistance to platinum, upregulation of miR-125b, miR-133a, miR-141, miR-182, miR-193b, miR-1307, miR-20a, miR-21, miR-27a, miR-215, miR-30a-5p, miR-31, miR-93, and miR-509-3p was related to cisplatin resistance, as was downregulation of miR-152, miR-199a, and let-7c. It is known that decreased resistance to platinum agents is related to upregulation of miR-149, miR-155, miR152, miR-199a, miR200b, miR-200c, miR-30d, miR-34c, miR-363, miR-497, miR-506, miR-9, and let-7i, and to downregulation of miR-23a and miR-603. There were discrepancies with miR-106a, miR-130b and miR-34a in terms of increased or decreased resistance to cisplatin when upregulated or downregulated (Supplementary Table 4).

In terms of resistance to paclitaxel, increased resistance was associated with upregulation of miR-106a, miR-182, miR-1307, miR-21, miR-27a, miR-30a and miR-490-3p and with downregulation of miR-141, miR-145, miR-148a, miR-149, and miR-200c. Decreased resistance to paclitaxel was associated with upregulation of miR-29b, let-7i, miR-199a, miR-200a, miR-200c and miR-215. There was also discrepancy in terms of increased sensitivity to paclitaxel and miR-130b expression (Supplementary Table 5).

### Overall survival meta-analysis

**Figure 4:**
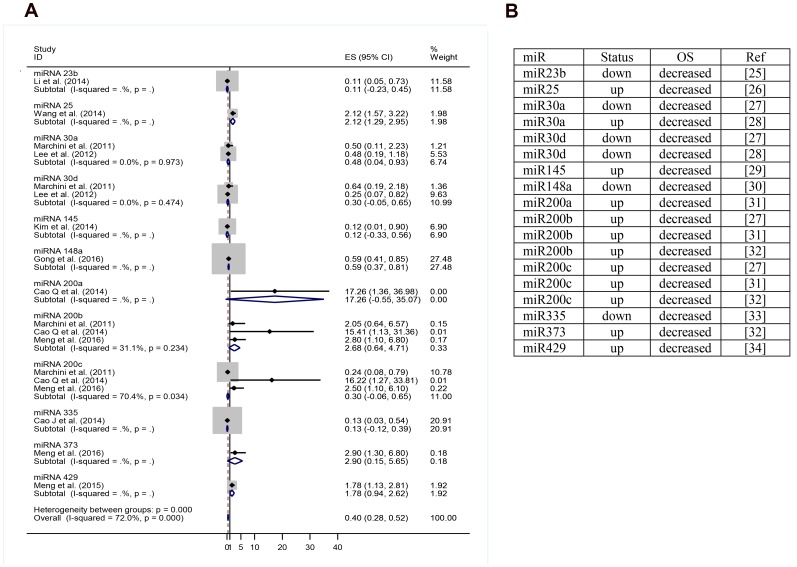
(**A**) miRNA expression level decreased versus increased expression level correlated to overall survival. (**B**) miRNA and their respective status correlated to overall survival. OS: overall survival; up: upregulation; down: downregulation; miR: miRNA; ref: reference.

**Figure 5:**
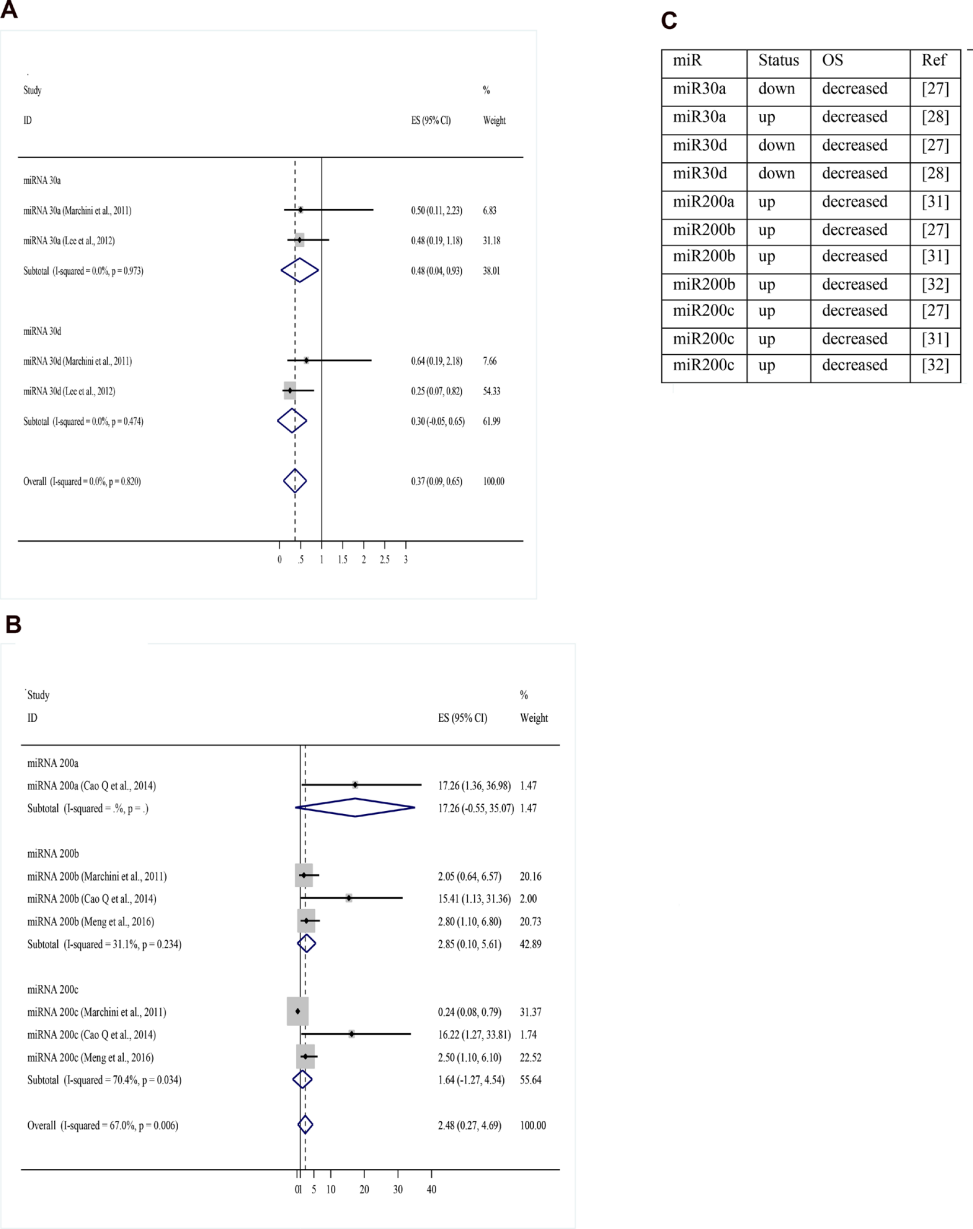
(**A**) Overall survival meta-analysis for miR-30 family (miRNA expression level decreased versus increased expression level). (**B**) Overall survival meta-analysis for miR-200 family (miRNA expression level decreased versus increased expression level). (**C**) Articles references of miRNA and their respective status correlated to OS. OS: overall survival; up: upregulation; down: downregulation; miR: miRNA; ref: reference.

For construction of the OS meta-analysis were selected miRNAs that had been cited three or more times. Two hundred and ninety miRNAs had been excluded for don’t addresses any OS data, leaving 60 miRNAs, 37 of which were excluded because they didn’t directly address OS. Eleven more were excluded for lacking necessary data. Twelve miRNAs were included in the final forest plot (Figure 4A and 4B). To miR-200 family, the meta-analysis results showed that when upregulated miR200b and miR-200a may signify risk factors related to decreased OS. Regarding miR-200c, results were inconclusive. In one article it was related to increased OS and in two other articles with decreased OS. To miR-30 family, miR-30a and miR-30d when upregulated, there appeared to be a trend towards increased OS, but it was not statistically significant It was found only one article for miR-145, miR-148a, miR-335, and miR-23b that when upregulated appear to indicate good prognosis. To miR-25, miR-429 and miR-373 was found only one article for miR and when upregulated may signify risk factors related to decreased OS. For the random effect model considering as weight of the studies the sample size was performed a test of heterogeneity: χ^2^ = 60.74 *p <* 0.001. I^2^ = 72.0%. (Figures 4A, 5A–5C).

## DISCUSSION

miRNAs have been shown to affect virtually all cellular functions including proliferation, apoptosis, cell cycle and differentiation [4], and they can act as either oncogenes or tumor suppressor genes. The expression profiles of miRNA in different tissues, blood-based serum, and plasma has also been thoroughly evaluated and may be used as surrogate prognostic biomarkers. Taylor and cols. compared the miRNA expression profile of serum and tumor-derived exosomes from the same patients and identified eight miRNAs (miR-21, miR141, miR-200a, miR-200b, miR-200c, miR-203, miR-205, and miR-214) with similar expression levels in both tissue and tumor-derived exosomes [5]. Circulating miRNAs could potentially serve as noninvasive diagnostic markers; in recent years, increasing amounts of research have led to rapid development of this field [5]. However, miRNA profiling studies have failed to show agreement between their results for some miRNAs. In this systematic review we focus on the most cited miRNAs in the literature to illustrate the many disagreements between studies. Further discussion of the present findings will focus on two major miRNA families: miR-200 and let-7.

### miRNAs as diagnostic biomarkers

Detection of ovarian cancer in its early stages is rare, but critical to effective treatment. Biomarkers with high sensitivity and specificity are urgently needed [5]. Iorio and cols. reported that different miRNA expression profiles could clearly differentiate ovarian cancer from normal controls [6]. Chen and cols. through systematic review postulated that upregulation of miR-200a, miR-200b, miR-200c and miR-141 and downregulation of miR-100 would be promising candidate biomarkers for epithelial ovarian cancer [7]. The miR-200 family includes miR-200a, miR-200b, miR-200c, miR-141, and miR-429, which are arranged in two clusters in the human genome. miR-200a, miR-200b and miR-429 are located on chromosome 1, while miR-200c and miR-141 are on chromosome 12 [8]. Iorio and cols. have shown that the miR-200 family is among the most significantly overexpressed miRNAs in epithelial ovarian cancer [6]. Expression levels of miR-200a and miR-200c were upregulated in three types of ovarian cancer: serous, endometrioid, and clear cell. However, miR-200b and miR-141 were upregulated in endometrioid and serous ovarian cancer [6].

The role of the miR-200 family in ovarian carcinoma is not completely understood. While it is believed to suppress metastasis, most studies done on the family show that it is overexpressed in ovarian cancer [9]. However, some studies have reported that miR-200 family members are either downregulated [9] or unchanged [10]. These conflicting results may be due to different normal controls or the inclusion of ovarian stromal cells which lack miR-expression. The most prominent targets of the miR-200 family are two binding transcription factors, ZEB1 and ZEB2, key regulators of a complex network of transcriptional repressors regulating E-cadherin expression and epithelial polarity [7]. ZEB1 and ZEB2 are involved in mediating the epithelial to mesenchymal transition and can inhibit the expression of miR-200 family members by binding to the promoter of both miR-200 clusters thereby blocking transcription [11]. Leva Di G. described how cancer cells, after being triggered by molecular signaling, increase their levels of ZEB1/2, which in turn decrease the expression of miR-200 and induce epithelial to mesenchymal transition [12].

In contrast, overexpression of miR-200 family members represses ZEB1/2, leading to higher levels of E-cadherin and an epithelial phenotype [13]. In fact, Park and cols. have shown a positive correlation between E-cadherin and miR-200c expression in ovarian cancer tissue. Low expression of the let-7 family has also been identified as a potential early marker for diagnosis [14–15]. The let-7 family in humans consists of 13 miRNAs located on nine different chromosomes [16–17]. In multiple human cancers expression of the let-7 family is known to be significantly reduced.

### miRNAs as prognostic biomarkers

Low expression of members of the let-7 family has also been associated with decreased overall survival in several studies. Most members of let-7 family are reported to act as tumor suppressors [14, 15]. Low let-7 expression has been associated with poor survival of cancer patients [18, 19]. let-7 suppresses multiple ovarian cancer oncogenes including KRAS, HRAS, c-MYC, and HMGA-2 [20]. It also inhibits cell cycle regulators such as CDC25, CDK6, and Cyclins A, D1, D2, and D3 [12, 13]. Cao and cols. showed that patients with high expression of miR-200a, miR-200b, and miR-200c present shorter OS than controls (all *p <* 0.001) [21]. Nam and cols. also associated high expression of the miR-200 family with decreased progression free survival (PFS) and OS of ovarian cancer patients [8]. Eitan and cols., however, reported that the expression levels of miR-200a and miR-200b may be lower in late than in early stage tumors, despite the fact that a high level of miR-200a expression in advanced stages has been correlated with poor ovarian cancer outcome [22, 23].

### miRNAs as chemotherapy response predictors

Recent studies have shown a correlation between loss of let-7 and resistance to chemotherapy [14, 15]. Leskelä and cols. showed that the miR-200 family (miR-141, miR-200a, miR-200b, miR-200c, and miR-429) is implicated in the response to paclitaxel treatment and PFS via regulation of β-tubulin III. MiR-200c is significantly associated with recurrence of ovarian cancer and miR-429 is associated with progression free and OS rates. Downregulation of miR-200 family causes increased expression of β-tubulin III, which leads to the development of chemoresistance in ovarian cancer patients. Cao and cols. (2014) concluded that patients with high expression of miR-200a, miR-200b, and mi-R200c present shorter overall survival than the corresponding controls (all *p <* 0.001) [21]. Moreover, Pal and cols. (2015) have shown that reduced expression of miR-200c is positively correlated with recurrence of ovarian cancer [22]. In addition, miR-200 overexpression significantly inhibits ovarian cancer cell invasiveness and metastasis by downregulating MMP3, possibly through ZEB1/pSMAD3 signaling [22].

### Overall survival meta-analysis

In our systematic review were found 72 review articles and only four systematic reviews with meta-analysis. Sun and cols. evaluated the prognostic value of miR-9 in different types of carcinomas. The study evaluated 16 articles and only three included ovarian cancer. They found that downregulation of miR-9 was associated with worse OS in ovarian cancer. The other types of cancer presented with opposite results: worse prognosis was associated with upregulation of miR-9. Liang *et al*. performed a meta-analysis evaluating miR-145 as a novel biomarker in ovarian cancer. Initially, the study compared the level of miR-145 between 135 healthy controls, 84 patients with malignant ovarian cancer, and 51 with benign ovarian cancer. The results showed that the relative expression of serum miR-145 was significantly downregulated in patients with malignant ovarian cancer and benign ovarian cancer compared to healthy controls (*p <* 0.01). Serum miR-145 level could discriminate patients with malignant ovarian cancer compared to healthy controls with a power area under the curve AUC of 0.82 (95% confidence interval +0.77-0.88). Furthermore, patients with low serum levels of miR-145 had significantly shorter median OS. In the same study, the authors performed a meta-analysis with the intention of evaluating the overall diagnostic accuracy of miR-145 for various cancer histology. Fifteen studies were included in the meta-analysis and the carcinomas included were bladder, biliary tract, prostate, colorectal, breast, lung, and pleural mesothelioma. Due to heterogeneity between studies, further analysis was conducted based on ethnicity and, compared to Asian populations, miR-145 exhibited a relatively higher overall diagnostic accuracy in Caucasian populations. Wang and cols. evaluated the apparent discrepancy between miR-200c expression and survival in solid tumors. It included in its analysis 5 studies including patients with gastric, endometrial, pancreatic, colorectal, and ovarian cancers. For construction of the forest plots the miRNAs were divided into serum miR-200c (two studies) and tissue miR-200c (two studies) and were correlated to overall survival. Results showed an increased mortality in patients with high expression of miR-200c in serum. In contrast, low expression of miR-200c in tissue indicated higher mortality.

Shi and cols. conducted the first meta-analysis of ovarian cancer alone and aimed to evaluate the prognostic molecular signature of miRNA-200 and miR-30 families. The author concluded that higher expression of miR-200c in tissue was significantly associated with better OS, and upregulation of miR-200c in blood was related with poorer OS. Meta-analysis of the miR-30 family revealed that elevated levels of miR-30a and miR-30d were associated with better OS. In our study, miR-200c in one article was identified as an indicator of good prognosis and in the other two articles as a high-risk factor. Results regarding miR-30a and miR-30d were inconclusive to OS, but with a trend toward being a good prognosis factor when upregulated.

Hu and cols. evaluated the prognostic value of miRNAs by expression profiling in 55 ovarian cancer patients. In this study, miR-200a showed a significant association with cancer survival, and overexpression of miR-200a predicted favorable disease outcome among 96 miRNAs analyzed [23]. However, Nam and cols. in another study showed that overexpression of miR-200a was associated with poor prognosis [24]. The results in most studies for the miR-200 family showed poor prognosis when miR-200a, miR-200b, and miR-429 were upregulated, and presented contradiction in the studies for miR-200c with a tendency to poor prognosis when upregulated. Regarding the let-7 family, the eligible studies didn’t include the necessary data to be included in the forest plot.

The present meta-analysis had some limitations. The studies demonstrated significant heterogeneity in sample size, follow-up length, and methodology for miRNA identification. Despite these differences, the intention of this systematic review was to identify the most mentioned miRNAs in literature focusing on prognostic and diagnostic features. The OS meta-analysis identified 12 miRNA candidates as strong prognostic and predictive markers that should be further investigated in randomized prospective studies.

## CONCLUSIONS

miRNAs have proven to be promising tools in cancer management. Since their discovery, in 1997, they have been studied as prognostic, diagnostic, and response predictive biomarkers. This systematic review provides a comprehensive review of miRNA as predictors of chemotherapy response and prognostic markers. These findings may improve early diagnosis of ovarian cancer and predict response to chemotherapy. Results need to be validated in prospective randomized trials.

## SUPPLEMENTARY MATERIALS









## References

[1] Ferlay J, Colombet M, Soerjomataram I, Mathers C, Parkin DM, Piñeros M, Znaor A, Bray F Estimating the global cancer incidence and mortality in 2018: GLOBOCAN sources and methods. Int J Cancer. 2019; 144:1941–53. 10.1002/ijc.31937. 30350310

[2] Jemal A, Siegel R, Xu J, Ward E Cancer statistics, 2010. CA Cancer J Clin. 2010; 60:277–300. 10.3322/caac.20073. 20610543

[3] Beller U, Benedet JL, Creasman WT, Ngan HY, Quinn MA, Maisonneuve P, Pecorelli S, Odicino F, Heintz AP Carcinoma of the vagina. FIGO 26th Annual Report on the Results of Treatment in Gynecological Cancer. Int J Gynaecol Obstet. 2006; 95:S29–42. 10.1016/S0020-7292(06)60029-5. 17161165

[4] Prahm KP, Novotny GW, Høgdall C, Høgdall E Current status on microRNAs as biomarkers for ovarian cancer. APMIS. 2016; 124:337–55. 10.1111/apm.12514. 26809719

[5] Zhao L, Wang W, Xu L, Yi T, Zhao X, Wei Y, Vermeulen L, Goel A, Zhou S, Wang X Integrative network biology analysis identifies miR-508-3p as the determinant for the mesenchymal identity and a strong prognostic biomarker of ovarian cancer. Oncogene. 2019; 38:2305–19. 10.1038/s41388-018-0577-5. 30478449 PMC6755993

[6] Zhang S, Zhang JY, Lu LJ, Wang CH, Wang LH MiR-630 promotes epithelial ovarian cancer proliferation and invasion via targeting KLF6. Eur Rev Med Pharmacol Sci. 2017; 21:4542–47. 29131262

[7] Zhan Y, Xiang F, Wu R, Xu J, Ni Z, Jiang J, Kang X MiRNA-149 modulates chemosensitivity of ovarian cancer A2780 cells to paclitaxel by targeting MyD88. J Ovarian Res. 2015; 8:48. 10.1186/s13048-015-0178-7. 26223974 PMC4520014

[8] Zou J, Yin F, Wang Q, Zhang W, Li L Analysis of microarray-identified genes and microRNAs associated with drug resistance in ovarian cancer. Int J Clin Exp Pathol. 2015; 8:6847–58. 26261572 PMC4525906

[9] Cao L, Wan Q, Li F, Tang CE MiR-363 inhibits cisplatin chemoresistance of epithelial ovarian cancer by regulating snail-induced epithelial-mesenchymal transition. BMB Rep. 2018; 51:456–61. 10.5483/BMBRep.2018.51.9.104. 30037365 PMC6177509

[10] Li Y, Yao L, Liu F, Hong J, Chen L, Zhang B, Zhang W Characterization of microRNA expression in serous ovarian carcinoma. Int J Mol Med. 2014; 34:491–98. 10.3892/ijmm.2014.1813. 24939816

[11] Chen Y, Zhang L, Hao Q Candidate microRNA biomarkers in human epithelial ovarian cancer: systematic review profiling studies and experimental validation. Cancer Cell Int. 2013; 13:86. 10.1186/1475-2867-13-86. 23978303 PMC3765519

[12] Leskelä S, Leandro-García LJ, Mendiola M, Barriuso J, Inglada-Pérez L, Muñoz I, Martínez-Delgado B, Redondo A, de Santiago J, Robledo M, Hardisson D, Rodríguez-Antona C The miR-200 family controls β-tubulin III expression and is associated with paclitaxel-based treatment response and progression-free survival in ovarian cancer patients. Endocr Relat Cancer. 2010; 18:85–95. 10.1677/ERC-10-0148. 21051560

[13] Nam EJ, Yoon H, Kim SW, Kim H, Kim YT, Kim JH, Kim JW, Kim S MicroRNA expression profiles in serous ovarian carcinoma. Clin Cancer Res. 2008; 14:2690–95. 10.1158/1078-0432.CCR-07-1731. 18451233

[14] Chen WT, Yang YJ, Zhang ZD, An Q, Li N, Liu W, Yang B MiR-1307 promotes ovarian cancer cell chemoresistance by targeting the ING5 expression. J Ovarian Res. 2017; 10:1. 10.1186/s13048-016-0301-4. 28086946 PMC5234104

[15] Zhou Y, Wang M, Wu J, Jie Z, Chang S, Shuang T The clinicopathological significance of miR-1307 in chemotherapy resistant epithelial ovarian cancer. J Ovarian Res. 2015; 8:23. 10.1186/s13048-015-0143-5. 25887170 PMC4449560

[16] Zou J, Liu L, Wang Q, Yin F, Yang Z, Zhang W, Li L Downregulation of miR-429 contributes to the development of drug resistance in epithelial ovarian cancer by targeting ZEB1. Am J Transl Res. 2017; 9:1357–68. 28386361 PMC5376026

[17] Eoh KJ, Lee SH, Kim HJ, Lee JY, Kim S, Kim SW, Kim YT, Nam EJ MicroRNA-630 inhibitor sensitizes chemoresistant ovarian cancer to chemotherapy by enhancing apoptosis. Biochem Biophys Res Commun. 2018; 497:513–20. 10.1016/j.bbrc.2018.02.062. 29452092

[18] Taylor DD, Gercel-Taylor C MicroRNA signatures of tumor-derived exosomes as diagnostic biomarkers of ovarian cancer. Gynecol Oncol. 2008; 110:13–21. 10.1016/j.ygyno.2008.04.033. 18589210

[19] Esquela-Kerscher A, Slack FJ Oncomirs - microRNAs with a role in cancer. Nat Rev Cancer. 2006; 6:259–69. 10.1038/nrc1840. 16557279

[20] Iorio MV, Visone R, Di Leva G, Donati V, Petrocca F, Casalini P, Taccioli C, Volinia S, Liu CG, Alder H, Calin GA, Ménard S, Croce CM MicroRNA signatures in human ovarian cancer. Cancer Res. 2007; 67:8699–707. 10.1158/0008-5472.CAN-07-1936. 17875710

[21] Wang Z, Ting Z, Li Y, Chen G, Lu Y, Hao X microRNA-199a is able to reverse cisplatin resistance in human ovarian cancer cells through the inhibition of mammalian target of rapamycin. Oncol Lett. 2013; 6:789–94. 10.3892/ol.2013.1448. 24137412 PMC3789061

[22] Marzec-Kotarska B, Cybulski M, Kotarski JC, Ronowicz A, Tarkowski R, Polak G, Antosz H, Piotrowski A, Kotarski J Molecular bases of aberrant miR-182 expression in ovarian cancer. Genes Chromosomes Cancer. 2016; 55:877–89. 10.1002/gcc.22387. 27295517

[23] Yuan JM, Shi XJ, Sun P, Liu JX, Wang W, Li M, Ling FY Downregulation of cell cycle-related proteins in ovarian cancer line and cell cycle arrest induced by microRNA. Int J Clin Exp Med. 2015; 8:18476–81. 26770455 PMC4694355

[24] Zhao X, Zhou Y, Chen YU, Yu F miR-494 inhibits ovarian cancer cell proliferation and promotes apoptosis by targeting FGFR2. Oncol Lett. 2016; 11:4245–51. 10.3892/ol.2016.4527. 27313773 PMC4888167

[25] Su L, Liu M Correlation analysis on the expression levels of microRNA-23a and microRNA-23b and the incidence and prognosis of ovarian cancer. Oncol Lett. 2018; 16:262–66. 10.3892/ol.2018.8669. 29928410 PMC6006491

[26] Hu X, Macdonald DM, Huettner PC, Feng Z, El Naqa IM, Schwarz JK, Mutch DG, Grigsby PW, Powell SN, Wang X A miR-200 microRNA cluster as prognostic marker in advanced ovarian cancer. Gynecol Oncol. 2009; 114:457–64. 10.1016/j.ygyno.2009.05.022. 19501389

[27] Marchini S, Cavalieri D, Fruscio R, Calura E, Garavaglia D, Fuso Nerini I, Mangioni C, Cattoretti G, Clivio L, Beltrame L, Katsaros D, Scarampi L, Menato G, et al Association between miR-200c and the survival of patients with stage I epithelial ovarian cancer: a retrospective study of two independent tumour tissue collections. Lancet Oncol. 2011; 12:273–85. 10.1016/S1470-2045(11)70012-2. 21345725

[28] Lee H, Park CS, Deftereos G, Morihara J, Stern JE, Hawes SE, Swisher E, Kiviat NB, Feng Q MicroRNA expression in ovarian carcinoma and its correlation with clinicopathological features. World J Surg Oncol. 2012; 10:174. 10.1186/1477-7819-10-174. 22925189 PMC3449188

[29] Kim TH, Song JY, Park H, Jeong JY, Kwon AY, Heo JH, Kang H, Kim G, An HJ miR-145, targeting high-mobility group A2, is a powerful predictor of patient outcome in ovarian carcinoma. Cancer Lett. 2015; 356:937–45. 10.1016/j.canlet.2014.11.011. 25444913

[30] Gong L, Wang C, Gao Y, Wang J Decreased expression of microRNA-148a predicts poor prognosis in ovarian cancer and associates with tumor growth and metastasis. Biomed Pharmacother. 2016; 83:58–63. 10.1016/j.biopha.2016.05.049. 27470550

[31] Cao Q, Lu K, Dai S, Hu Y, Fan W Clinicopathological and prognostic implications of the miR-200 family in patients with epithelial ovarian cancer. Int J Clin Exp Pathol. 2014; 7:2392–401. 24966949 PMC4069884

[32] Meng X, Müller V, Milde-Langosch K, Trillsch F, Pantel K, Schwarzenbach H Diagnostic and prognostic relevance of circulating exosomal miR-373, miR-200a, miR-200b and miR-200c in patients with epithelial ovarian cancer. Oncotarget. 2016; 7:16923–35. 10.18632/oncotarget.7850. 26943577 PMC4941360

[33] Cao J, Cai J, Huang D, Han Q, Chen Y, Yang Q, Yang C, Kuang Y, Li D, Wang Z miR-335 represents an independent prognostic marker in epithelial ovarian cancer. Am J Clin Pathol. 2014; 141:437–42. 10.1309/AJCPLYTZGB54ISZC. 24515774

[34] Meng X, Joosse SA, Müller V, Trillsch F, Milde-Langosch K, Mahner S, Geffken M, Pantel K, Schwarzenbach H Diagnostic and prognostic potential of serum miR-7, miR-16, miR-25, miR-93, miR-182, miR-376a and miR-429 in ovarian cancer patients. Br J Cancer. 2015; 113:1358–66. 10.1038/bjc.2015.340. 26393886 PMC4815782

