# Supplementary material for: The prognostic role of microRNA in epithelial ovarian cancer: a systematic review of literature with an overall survival meta-analysis
Source: Oncotarget. 2020 Mar 24;11(12):1085–95. doi: 10.18632/oncotarget.27246 (PMC7105164; doi:10.18632/oncotarget.27246)
Supplement: Supplementary file 1 [file oncotarget-11-1085-s001.pdf]

The prognostic role of microRNA in epithelial ovarian cancer: a systematic review of literature with an overall survival meta-analysis

SUPPLEMENTARY MATERIALS

REFERENCES

1. Kim YW, Kim EY, Jeon D, Liu JL, Kim HS, Choi JW, Ahn WS. Differential microRNA expression signatures and cell type-specific association with Taxol resistance in ovarian cancer cells. Drug Des Devel Ther. 2014; 8:293–314. https://doi.org/10.2147/DDDT.S51969. [PubMed]
2. Huh JH, Kim TH, Kim K, Song JA, Jung YJ, Jeong JY, Lee MJ, Kim YK, Lee DH, An HJ. Dysregulation of miR-106a and miR-591 confers paclitaxel resistance to ovarian cancer. Br J Cancer. 2013; 109:452–61. https://doi.org/10.1038/bjc.2013.305. [PubMed]
3. Chen L, Zhang F, Sheng XG, Zhang SQ, Chen YT, Liu BW. MicroRNA-106a regulates phosphatase and tensin homologue expression and promotes the proliferation and invasion of ovarian cancer cells. Oncol Rep. 2016; 36:2135–41. https://doi.org/10.3892/or.2016.5010. [PubMed]
4. Li H, Xu H, Shen H, Li H. microRNA-106a modulates cisplatin sensitivity by targeting PDCD4 in human ovarian cancer cells. Oncol Lett. 2014; 7:183–88. https://doi.org/10.3892/ol.2013.1644 [PubMed]
5. Rao YM, Shi HR, Ji M, Chen CH. MiR-106a targets Mcl-1 to suppress cisplatin resistance of ovarian cancer A2780 cells. J Huazhong Univ Sci Technolog Med Sci. 2013; 33:567–72. Medical Sciences https://doi.org/10.1007/s11596-013-1160-5. [PubMed]
6. Ying X, Wei K, Lin Z, Cui Y, Ding J, Chen Y, Xu B. Microrna-125b suppresses ovarian cancer progression via suppression of the epithelial-mesenchymal transition pathway by targeting the set protein. Cell Physiol Biochem. 2016; 39:501–10. https://doi.org/10.1159/000445642. [PubMed]
7. Liu M, Zhang X, Hu CF, Xu Q, Zhu HX, Xu NZ. MicroRNA-mRNA functional pairs for cisplatin resistance in ovarian cancer cells. Chin J Cancer. 2014; 33:285–94. https://doi.org/10.5732/cjc.013.10136. [PubMed]
8. Lee M, Kim EJ, Jeon MJ. MicroRNAs 125a and 125b inhibit ovarian cancer cells through post-transcriptional inactivation of EIF4EBP1. Oncotarget. 2016; 7:8726–42. https://doi.org/10.18632/oncotarget.6474. [PubMed]
9. Guan Y, Yao H, Zheng Z, Qiu G, Sun K. MiR-125b targets BCL3 and suppresses ovarian cancer proliferation. Int J Cancer. 2011; 128:2274–83. https://doi.org/10.1002/ijc.25575. [PubMed]
10. Luo S, Wang J, Ma Y, Yao Z, Pan H. PPARγ inhibits ovarian cancer cells proliferation through upregulation of miR-125b. Biochem Biophys Res Commun. 2015; 462:85–90. https://doi.org/10.1016/j.bbrc.2015.04.023. [PubMed]
11. He J, Xu Q, Jing Y, Agani F, Qian X, Carpenter R, Li Q, Wang XR, Peiper SS, Lu Z, Liu LZ, Jiang BH. Reactive oxygen species regulate ERBB2 and ERBB3 expression via miR-199a/125b and DNA methylation. EMBO Rep. 2012; 13:1116–22. https://doi.org/10.1038/embor.2012.162. [PubMed]
12. He J, Jing Y, Li W, Qian X, Xu Q, Li FS, Liu LZ, Jiang BH, Jiang Y. Roles and mechanism of miR-199a and miR-125b in tumor angiogenesis. PLoS One. 2013; 8:e56647. https://doi.org/10.1371/journal.pone.0056647. [PubMed]
13. Shahab SW, Matyunina LV, Hill CG, Wang L, Mezencev R, Walker LD, McDonald JF. The effects of MicroRNA transfections on global patterns of gene expression in ovarian cancer cells are functionally coordinated. BMC Med Genomics. 2012; 5:33. https://doi.org/10.1186/1755-8794-5-33. [PubMed]
14. Yang C, Cai J, Wang Q, Tang H, Cao J, Wu L, Wang Z. Epigenetic silencing of miR-130b in ovarian cancer promotes the development of multidrug resistance by targeting colony-stimulating factor 1. Gynecol Oncol. 2012; 124:325–34. https://doi.org/10.1016/j.ygyno.2011.10.013. [PubMed]
15. Zong C, Wang J, Shi TM. MicroRNA 130b enhances drug resistance in human ovarian cancer cells. Tumour Biol. 2014; 35:12151–56. https://doi.org/10.1007/s13277-014-2520-x. [PubMed]
16. Paudel D, Zhou W, Ouyang Y, Dong S, Huang Q, Giri R, Wang J, Tong X. MicroRNA-130b functions as a tumor suppressor by regulating RUNX3 in epithelial ovarian cancer. Gene. 2016; 586:48–55. https://doi.org/10.1016/j.gene.2016.04.001. [PubMed]
17. Guo J, Xia B, Meng F, Lou G. miR-133a suppresses ovarian cancer cell proliferation by directly targeting insulin-like growth factor 1 receptor. Tumour Biol. 2014; 35:1557–64. https://doi.org/10.1007/s13277-013-1215-z. [PubMed]
18. Luo J, Zhou J, Cheng Q, Zhou C, Ding Z. Role of microRNA-133a in epithelial ovarian cancer pathogenesis and progression. Oncol Lett. 2014; 7:1043–48. https://doi.org/10.3892/ol.2014.1841. [PubMed]

19. Zhang L, Li Z, Gai F, Wang Y. MicroRNA-137 suppresses tumor growth in epithelial ovarian cancer in vitro and in vivo. Mol Med Rep. 2015; 12:3107–14. https://doi.org/10.3892/mmr.2015.3756. [PubMed]
20. Dong P, Xiong Y, Watari H, Hanley SJ, Konno Y, Ihira K, Yamada T, Kudo M, Yue J, Sakuragi N. MiR-137 and miR-34a directly target Snail and inhibit EMT, invasion and sphere-forming ability of ovarian cancer cells. J Exp Clin Cancer Res. 2016; 35:132. https://doi.org/10.1186/s13046-016-0415-y. [PubMed]
21. Guo J, Xia B, Meng F, Lou G. miR-137 suppresses cell growth in ovarian cancer by targeting AEG-1. Biochem Biophys Res Commun. 2013; 441:357–63. https://doi.org/10.1016/j.bbrc.2013.10.052. [PubMed]
22. Wang Y, Li J, Xu C, Zhang X. MicroRNA-139-5p Inhibits Cell Proliferation and Invasion by Targeting RHO-Associated Coiled-Coil-Containing Protein Kinase 2 in Ovarian Cancer. Oncol Res. 2018; 26:411–20. https://doi.org/10.3727/096504017X14974343584989. [PubMed]
23. Li Y, Yao L, Liu F, Hong J, Chen L, Zhang B, Zhang W. Characterization of microRNA expression in serous ovarian carcinoma. Int J Mol Med. 2014; 34:491–98. https://doi.org/10.3892/ijmm.2014.1813. [PubMed]
24. Su YY, Sun L, Guo ZR, Li JC, Bai TT, Cai XX, Li WH, Zhu YF. Upregulated expression of serum exosomal miR-375 and miR-1307 enhance the diagnostic power of CA125 for ovarian cancer. J Ovarian Res. 2019; 12:6. https://doi.org/10.1186/s13048-018-0477-x. [PubMed]
25. Zou J, Yin F, Wang Q, Zhang W, Li L. Analysis of microarray-identified genes and microRNAs associated with drug resistance in ovarian cancer. Int J Clin Exp Pathol. 2015; 8:6847–58. [PubMed]
26. Iorio MV, Visone R, Di Leva G, Donati V, Petrocca F, Casalini P, Taccioli C, Volinia S, Liu CG, Alder H, Calin GA, Ménard S, Croce CM. MicroRNA signatures in human ovarian cancer. Cancer Res. 2007; 67:8699–707. https://doi.org/10.1158/0008-5472.CAN-07-1936. [PubMed]
27. Mateescu B, Batista L, Cardon M, Gruosso T, de Feraudy Y, Mariani O, Nicolas A, Meyniel JP, Cottu P, Sastre-Garau X, Mechta-Grigoriou F. miR-141 and miR-200a act on ovarian tumorigenesis by controlling oxidative stress response. Nat Med. 2011; 17:1627–35. https://doi.org/10.1038/nm.2512. [PubMed]
28. van Jaarsveld MT, Helleman J, Boersma AW, van Kuijk PF, van Ijcken WF, Despierre E, Vergote I, Mathijssen RH, Berns EM, Verweij J, Pothof J, Wiemer EA. miR-141 regulates KEAP1 and modulates cisplatin sensitivity in ovarian cancer cells. Oncogene. 2013; 32:4284–93. https://doi.org/10.1038/onc.2012.433. [PubMed]
29. Brozovic A, Duran GE, Wang YC, Francisco EB, Sikic BI. The miR-200 family differentially regulates sensitivity to paclitaxel and carboplatin in human ovarian carcinoma OVCAR-3 and MES-OV cells. Mol Oncol. 2015; 9:1678–93. https://doi.org/10.1016/j.molonc.2015.04.015. [PubMed]
30. Lam SS, Ip CK, Mak AS, Wong AS. A novel p70 S6 kinase-microRNA biogenesis axis mediates multicellular spheroid formation in ovarian cancer progression. Oncotarget. 2016; 7:38064–77. https://doi.org/10.18632/oncotarget.9345. [PubMed]
31. Zhang W, Wang Q, Yu M, Wu N, Wang H. MicroRNA-145 function as a cell growth repressor by directly targeting c-Myc in human ovarian cancer. Technol Cancer Res Treat. 2014; 13:161–68. https://doi.org/10.7785/tcrt.2012.500367. [PubMed]
32. Chen X, Dong C, Law PT, Chan MT, Su Z, Wang S, Wu WK, Xu H. MicroRNA-145 targets TRIM2 and exerts tumor-suppressing functions in epithelial ovarian cancer. Gynecol Oncol. 2015; 139:513–19. https://doi.org/10.1016/j.ygyno.2015.10.008. [PubMed]
33. Dong R, Liu X, Zhang Q, Jiang Z, Li Y, Wei Y, Li Y, Yang Q, Liu J, Wei JJ, Shao C, Liu Z, Kong B. miR-145 inhibits tumor growth and metastasis by targeting metadherin in high-grade serous ovarian carcinoma. Oncotarget. 2014; 5:10816–29. https://doi.org/10.18632/oncotarget.2522. [PubMed]
34. Wu H, Xiao Z, Wang K, Liu W, Hao Q. MiR-145 is downregulated in human ovarian cancer and modulates cell growth and invasion by targeting p70S6K1 and MUC1. Biochem Biophys Res Commun. 2013; 441:693–700. https://doi.org/10.1016/j.bbrc.2013.10.053. [PubMed]
35. Zhu X, Li Y, Xie C, Yin X, Liu Y, Cao Y, Fang Y, Lin X, Xu Y, Xu W, Shen H, Wen J. miR-145 sensitizes ovarian cancer cells to paclitaxel by targeting Sp1 and Cdk6. Int J Cancer. 2014; 135:1286–96. https://doi.org/10.1002/ijc.28774. [PubMed]
36. Kim TH, Song JY, Park H, Jeong JY, Kwon AY, Heo JH, Kang H, Kim G, An HJ. miR-145, targeting high-mobility group A2, is a powerful predictor of patient outcome in ovarian carcinoma. Cancer Lett. 2015; 356:937–45. https://doi.org/10.1016/j.canlet.2014.11.011. [PubMed]
37. Zhou X, Zhao F, Wang ZN, Song YX, Chang H, Chiang Y, Xu HM. Altered expression of miR-152 and miR-148a in ovarian cancer is related to cell proliferation. Oncol Rep. 2012; 27:447–54. https://doi.org/10.3892/or.2011.1482. [PubMed]
38. Gong L, Wang C, Gao Y, Wang J. Decreased expression of microRNA-148a predicts poor prognosis in ovarian cancer and associates with tumor growth and metastasis. Biomed Pharmacother. 2016; 83:58–63. https://doi.org/10.1016/j.biopha.2016.05.049. [PubMed]
39. Wen Z, Zhao S, Liu S, Liu Y, Li X, Li S. MicroRNA-148a inhibits migration and invasion of ovarian cancer cells via targeting sphingosine-1-phosphate receptor 1. Mol Med Rep. 2015; 12:3775–80. https://doi.org/10.3892/mmr.2015.3827. [PubMed]
40. Zhao S, Wen Z, Liu S, Liu Y, Li X, Ge Y, Li S. MicroRNA-148a inhibits the proliferation and promotes the paclitaxel-induced apoptosis of ovarian cancer cells by targeting PDIA3. Mol Med Rep. 2015; 12:3923–29. https://doi.org/10.3892/mmr.2015.3826. [PubMed]

41. Sun L, Zhai R, Zhang L, Zhao S. MicroRNA-149 suppresses the proliferation and increases the sensitivity of ovarian cancer cells to cisplatin by targeting X-linked inhibitor of apoptosis. Oncol Lett. 2018; 15:7328–34. https://doi.org/10.3892/ol.2018.8240. [PubMed]
42. Prahm KP, Novotny GW, Høgdall C, Høgdall E. Current status on microRNAs as biomarkers for ovarian cancer. APMIS. 2016; 124:337–55. https://doi.org/10.1111/apm.12514. [PubMed]
43. Xiang Y, Ma N, Wang D, Zhang Y, Zhou J, Wu G, Zhao R, Huang H, Wang X, Qiao Y, Li F, Han D, Wang L, et al. MiR-152 and miR-185 co-contribute to ovarian cancer cells cisplatin sensitivity by targeting DNMT1 directly: a novel epigenetic therapy independent of decitabine. Oncogene. 2014; 33:378–86. https://doi.org/10.1038/onc.2012.575. [PubMed]
44. Woo HH, László CF, Greco S, Chambers SK. Regulation of colony stimulating factor-1 expression and ovarian cancer cell behavior in vitro by miR-128 and miR-152. Mol Cancer. 2012; 11:58. https://doi.org/10.1186/1476-4598-11-58. [PubMed]
45. Cubillos-Ruiz JR, Sempere LF, Conejo-Garcia JR. Good things come in small packages: therapeutic anti-tumor immunity induced by microRNA nanoparticles. Oncoimmunology. 2012; 1:968–70. https://doi.org/10.4161/onci.20020. [PubMed]
46. Cao L, Wan Q, Li F, Tang CE. MiR-363 inhibits cisplatin chemoresistance of epithelial ovarian cancer by regulating snail-induced epithelial-mesenchymal transition. BMB Rep. 2018; 51:456–61. https://doi.org/10.5483/BMBRep.2018.51.9.104. [PubMed]
47. Chen W, Huang L, Hao C, Zeng W, Luo X, Li X, Zhou L, Jiang S, Chen Z, He Y. MicroRNA-155 promotes apoptosis in SKOV3, A2780, and primary cultured ovarian cancer cells. Tumour Biol. 2016; 37:9289–99. https://doi.org/10.1007/s13277-016-4804-9. [PubMed]
48. Resnick KE, Alder H, Hagan JP, Richardson DL, Croce CM, Cohn DE. The detection of differentially expressed microRNAs from the serum of ovarian cancer patients using a novel real-time PCR platform. Gynecol Oncol. 2009; 112:55–59. https://doi.org/10.1016/j.ygyno.2008.08.036. [PubMed]
49. Xu X, Ayub B, Liu Z, Serna VA, Qiang W, Liu Y, Hernando E, Zabludoff S, Kurita T, Kong B, Wei JJ. Anti-miR182 reduces ovarian cancer burden, invasion, and metastasis: an in vivo study in orthotopic xenografts of nude mice. Mol Cancer Ther. 2014; 13:1729–39. https://doi.org/10.1158/1535-7163.MCT-13-0982. [PubMed]
50. McMillen BD, Aponte MM, Liu Z, Helenowski IB, Scholtens DM, Buttin BM, Wei JJ. Expression analysis of MIR182 and its associated target genes in advanced ovarian carcinoma. Mod Pathol. 2012; 25:1644–53. https://doi.org/10.1038/modpathol.2012.118. [PubMed]
51. Meng X, Joosse SA, Müller V, Trillsch F, Milde-Langosch K, Mahner S, Geffken M, Pantel K, Schwarzenbach H. Diagnostic and prognostic potential of serum miR-7, miR-16, miR-25, miR-93, miR-182, miR-376a and miR-429 in ovarian cancer patients. Br J Cancer. 2015; 113:1358–66. https://doi.org/10.1038/bjc.2015.340. [PubMed]
52. Wang YQ, Guo RD, Guo RM, Sheng W, Yin LR. MicroRNA-182 promotes cell growth, invasion, and chemoresistance by targeting programmed cell death 4 (PDCD4) in human ovarian carcinomas. J Cell Biochem. 2013; 114:1464–73. https://doi.org/10.1002/jcb.24488. [PubMed]
53. Liu Z, Liu J, Segura MF, Shao C, Lee P, Gong Y, Hernando E, Wei JJ. MiR-182 overexpression in tumourigenesis of high-grade serous ovarian carcinoma. J Pathol. 2012; 228:204–15. https://doi.org/10.1002/path.4000. [PubMed]
54. Marzec-Kotarska B, Cybulski M, Kotarski JC, Ronowicz A, Tarkowski R, Polak G, Antosz H, Piotrowski A, Kotarski J. Molecular bases of aberrant miR-182 expression in ovarian cancer. Genes Chromosomes Cancer. 2016; 55:877–89. https://doi.org/10.1002/gcc.22387. [PubMed]
55. Mitra AK, Chiang CY, Tiwari P, Tomar S, Watters KM, Peter ME, Lengyel E. Microenvironment-induced downregulation of miR-193b drives ovarian cancer metastasis. Oncogene. 2015; 34:5923–32. https://doi.org/10.1038/onc.2015.43. [PubMed]
56. Kumar S, Kumar A, Shah PP, Rai SN, Panguluri SK, Kakar SS. MicroRNA signature of cis-platin resistant vs. cis-platin sensitive ovarian cancer cell lines. J Ovarian Res. 2011; 4:17. https://doi.org/10.1186/1757-2215-4-17. [PubMed]
57. Wang Z, Ting Z, Li Y, Chen G, Lu Y, Hao X. microRNA-199a is able to reverse cisplatin resistance in human ovarian cancer cells through the inhibition of mammalian target of rapamycin. Oncol Lett. 2013; 6:789–94. https://doi.org/10.3892/ol.2013.1448. [PubMed]
58. Cheng W, Liu T, Wan X, Gao Y, Wang H. MicroRNA-199a targets CD44 to suppress the tumorigenicity and multidrug resistance of ovarian cancer-initiating cells. FEBS J. 2012; 279:2047–59. https://doi.org/10.1111/j.1742-4658.2012.08589.x. [PubMed]
59. Deng Y, Zhao F, Hui L, Li X, Zhang D, Lin W, Chen Z, Ning Y. Suppressing miR-199a-3p by promoter methylation contributes to tumor aggressiveness and cisplatin resistance of ovarian cancer through promoting DDR1 expression. J Ovarian Res. 2017; 10:50. https://doi.org/10.1186/s13048-017-0333-4. [PubMed]
60. Au Yeung CL, Co NN, Tsuruga T, Yeung TL, Kwan SY, Leung CS, Li Y, Lu ES, Kwan K, Wong KK, Schmandt R, Lu KH, Mok SC. Exosomal transfer of stroma-derived miR21 confers paclitaxel resistance in ovarian cancer cells through targeting APAF1. Nat Commun. 2016; 7:11150. https://doi.org/10.1038/ncomms11150. [PubMed]
61. Chen Y, Chen Q, Liu Q, Gao F. Human epididymis protein 4 expression positively correlated with miR-21 and served as a prognostic indicator in ovarian cancer. Tumour Biol. 2016; 37:8359–65. https://doi.org/10.1007/s13277-015-4672-8. [PubMed]
62. Xu YZ, Xi QH, Ge WL, Zhang XQ. Identification of serum microRNA-21 as a biomarker for early detection and

prognosis in human epithelial ovarian cancer. Asian Pac J Cancer Prev. 2013; 14:1057–60. https://doi.org/10.7314/APJCP.2013.14.2.1057. [PubMed]

63. d'Adhemar CJ, Spillane CD, Gallagher MF, Bates M, Costello KM, Barry-O'Crowley J, Haley K, Kernan N, Murphy C, Smyth PC, O'Byrne K, Pennington S, Cooke AA, et al. The MyD88+ phenotype is an adverse prognostic factor in epithelial ovarian cancer. PLoS One. 2014; 9:e100816. https://doi.org/10.1371/journal.pone.0100816. [PubMed]

64. Chan JK, Blansit K, Kiet T, Sherman A, Wong G, Earle C, Bourguignon LY. The inhibition of miR-21 promotes apoptosis and chemosensitivity in ovarian cancer. Gynecol Oncol. 2014; 132:739–44. https://doi.org/10.1016/j.ygyno.2014.01.034. [PubMed]

65. Mahmoud EH, Fawzy A, A Elshimy RA. Serum MicroRNA-21 Negatively Relates to Expression of Programmed Cell Death-4 in Patients with Epithelial Ovarian Cancer. Asian Pac J Cancer Prev. 2018; 19:33–38. https://doi.org/10.22034/APJCP.2018.19.1.33. [PubMed]

66. Li J, Liang S, Yu H, Zhang J, Ma D, Lu X. An inhibitory effect of miR-22 on cell migration and invasion in ovarian cancer. Gynecol Oncol. 2010; 119:543–48. https://doi.org/10.1016/j.ygyno.2010.08.034. [PubMed]

67. Ji T, Zheng ZG, Wang FM, Xu LJ, Li LF, Cheng QH, Guo JF, Ding XF. Differential microRNA expression by Solexa sequencing in the sera of ovarian cancer patients. Asian Pac J Cancer Prev. 2014; 15:1739–43. https://doi.org/10.7314/APJCP.2014.15.4.1739. [PubMed]

68. Wan WN, Zhang YQ, Wang XM, Liu YJ, Zhang YX, Que YH, Zhao WJ, Li P. Down-regulated miR-22 as predictive biomarkers for prognosis of epithelial ovarian cancer. Diagn Pathol. 2014; 9:178. https://doi.org/10.1186/s13000-014-0178-8. [PubMed]

69. Lenkala D, LaCroix B, Gamazon ER, Geeleher P, Im HK, Huang RS. The impact of microRNA expression on cellular proliferation. Hum Genet. 2014; 133:931–38. https://doi.org/10.1007/s00439-014-1434-4. [PubMed]

70. Wang X, Meng X, Li H, Liu W, Shen S, Gao Z. MicroRNA-25 expression level is an independent prognostic factor in epithelial ovarian cancer. Clin Transl Oncol. 2014; 16:954–58. https://doi.org/10.1007/s12094-014-1178-6. [PubMed]

71. Feng S, Pan W, Jin Y, Zheng J. MiR-25 promotes ovarian cancer proliferation and motility by targeting LATS2. Tumour Biol. 2014; 35:12339–44. https://doi.org/10.1007/s13277-014-2546-0. [PubMed]

72. Zhang H, Zuo Z, Lu X, Wang L, Wang H, Zhu Z. MiR-25 regulates apoptosis by targeting Bim in human ovarian cancer. Oncol Rep. 2012; 27:594–98. https://doi.org/10.3892/or.2011.1530. [PubMed]

73. Su L, Liu M. Correlation analysis on the expression levels of microRNA-23a and microRNA-23b and the incidence and prognosis of ovarian cancer. Oncol Lett. 2018; 16:262–66. https://doi.org/10.3892/ol.2018.8669. [PubMed]

74. Hu X, Macdonald DM, Huettner PC, Feng Z, El Naqa IM, Schwarz JK, Mutch DG, Grigsby PW, Powell SN, Wang X. A miR-200 microRNA cluster as prognostic marker in advanced ovarian cancer. Gynecol Oncol. 2009; 114:457–64. https://doi.org/10.1016/j.ygyno.2009.05.022. [PubMed]

75. Li Z, Hu S, Wang J, Cai J, Xiao L, Yu L, Wang Z. MiR-27a modulates MDR1/P-glycoprotein expression by targeting HIPK2 in human ovarian cancer cells. Gynecol Oncol. 2010; 119:125–30. https://doi.org/10.1016/j.ygyno.2010.06.004. [PubMed]

76. Bagnoli M, Canevari S, Califano D, Losito S, Maio MD, Raspagliesi F, Carcangiu ML, Toffoli G, Cecchin E, Sorio R, Canzonieri V, Russo D, Scognamiglio G, et al, and Multicentre Italian Trials in Ovarian cancer (MITO) translational group. Development and validation of a microRNA-based signature (MiROvaR) to predict early relapse or progression of epithelial ovarian cancer: a cohort study. Lancet Oncol. 2016; 17:1137–46. https://doi.org/10.1016/S1470-2045(16)30108-5. [PubMed]

77. Eitan R, Kushnir M, Lithwick-Yanai G, David MB, Hoshen M, Glezerman M, Hod M, Sabah G, Rosenwald S, Levavi H. Tumor microRNA expression patterns associated with resistance to platinum based chemotherapy and survival in ovarian cancer patients. Gynecol Oncol. 2009; 114:253–59. https://doi.org/10.1016/j.ygyno.2009.04.024. [PubMed]

78. Dai F, Zhang Y, Chen Y. Involvement of miR-29b signaling in the sensitivity to chemotherapy in patients with ovarian carcinoma. Hum Pathol. 2014; 45:1285–93. https://doi.org/10.1016/j.humpath.2014.02.008. [PubMed]

79. Teng Y, Zhang Y, Qu K, Yang X, Fu J, Chen W, Li X. MicroRNA-29B (mir-29b) regulates the Warburg effect in ovarian cancer by targeting AKT2 and AKT3. Oncotarget. 2015; 6:40799–814. https://doi.org/10.18632/oncotarget.5695. [PubMed]

80. Dai F, Zhang Y, Zhu X, Shan N, Chen Y. The anti-chemoresistant effect and mechanism of MUC1 aptamer-miR-29b chimera in ovarian cancer. Gynecol Oncol. 2013; 131:451–59. https://doi.org/10.1016/j.ygyno.2013.07.112. [PubMed]

81. Cao Q, Lu K, Dai S, Hu Y, Fan W. Clinicopathological and prognostic implications of the miR-200 family in patients with epithelial ovarian cancer. Int J Clin Exp Pathol. 2014; 7:2392–401. [PubMed]

82. Meng X, Müller V, Milde-Langosch K, Trillsch F, Pantel K, Schwarzenbach H. Diagnostic and prognostic relevance of circulating exosomal miR-373, miR-200a, miR-200b and miR-200c in patients with epithelial ovarian cancer. Oncotarget. 2016; 7:16923–35. https://doi.org/10.18632/oncotarget.7850. [PubMed]

83. Zuberi M, Mir R, Das J, Ahmad I, Javid J, Yadav P, Masroor M, Ahmad S, Ray PC, Saxena A. Expression of serum miR-200a, miR-200b, and miR-200c as candidate biomarkers in epithelial ovarian cancer and their association with clinicopathological features. Clin Transl Oncol. 2015; 17:779–87. https://doi.org/10.1007/s12094-015-1303-1. [PubMed]

84. Zhu CL, Gao GS. miR-200a overexpression in advanced ovarian carcinomas as a prognostic indicator. Asian Pac J Cancer Prev. 2014; 15:8595–601. https://doi.org/10.7314/APJCP.2014.15.20.8595. [PubMed]
85. Xu S, Xu P, Wu W, Ou Y, Xu J, Zhang G, Li J, Xu G. The biphasic expression pattern of miR-200a and E-cadherin in epithelial ovarian cancer and its correlation with clinicopathological features. Curr Pharm Des. 2014; 20:1888–95. https://doi.org/10.2174/13816128113199990523. [PubMed]
86. Liu N, Zhong L, Zeng J, Zhang X, Yang Q, Liao D, Wang Y, Chen G, Wang Y. Upregulation of microRNA-200a associates with tumor proliferation, CSCs phenotype and chemosensitivity in ovarian cancer. Neoplasma. 2015; 62:550–59. https://doi.org/10.4149/neo_2015_066. [PubMed]
87. Wu Q, Guo R, Lin M, Zhou B, Wang Y. MicroRNA-200a inhibits CD133/1+ ovarian cancer stem cells migration and invasion by targeting E-cadherin repressor ZEB2. Gynecol Oncol. 2011; 122:149–54. https://doi.org/10.1016/j.ygyno.2011.03.026. [PubMed]
88. Kapetanakis NI, Uzan C, Jimenez-Pailhes AS, Gouy S, Bentivegna E, Morice P, Caron O, Gourzones-Dmitriev C, Le Teuff G, Busson P. Plasma miR-200b in ovarian carcinoma patients: distinct pattern of pre/post-treatment variation compared to CA-125 and potential for prediction of progression-free survival. Oncotarget. 2015; 6:36815–24. https://doi.org/10.18632/oncotarget.5766. [PubMed]
89. Gao YC, Wu J. MicroRNA-200c and microRNA-141 as potential diagnostic and prognostic biomarkers for ovarian cancer. Tumour Biol. 2015; 36:4843–50. https://doi.org/10.1007/s13277-015-3138-3. [PubMed]
90. Cochrane DR, Spoelstra NS, Howe EN, Nordeen SK, Richer JK. MicroRNA-200c mitigates invasiveness and restores sensitivity to microtubule-targeting chemotherapeutic agents. Mol Cancer Ther. 2009; 8:1055–66. https://doi.org/10.1158/1535-7163.MCT-08-1046. [PubMed]
91. Lu YM, Shang C, Ou YL, Yin D, Li YN, Li X, Wang N, Zhang SL. miR-200c modulates ovarian cancer cell metastasis potential by targeting zinc finger E-box-binding homeobox 2 (ZEB2) expression. Med Oncol. 2014; 31:134. https://doi.org/10.1007/s12032-014-0134-1. [PubMed]
92. Lu L, Schwartz P, Scarampi L, Rutherford T, Canuto EM, Yu H, Katsaros D. MicroRNA let-7a: a potential marker for selection of paclitaxel in ovarian cancer management. Gynecol Oncol. 2011; 122:366–71. https://doi.org/10.1016/j.ygyno.2011.04.033. [PubMed]
93. Prislei S, Martinelli E, Mariani M, Raspaglio G, Sieber S, Ferrandina G, Shahabi S, Scambia G, Ferlini C. MiR-200c and HuR in ovarian cancer. BMC Cancer. 2013; 13:72. https://doi.org/10.1186/1471-2407-13-72. [PubMed]
94. Ibrahim FF, Jamal R, Syafruddin SE, Ab Mutalib NS, Saidin S, MdZin RR, Hossain Mollah MM, Mokhtar NM. MicroRNA-200c and microRNA-31 regulate proliferation, colony formation, migration and invasion in serous ovarian cancer. J Ovarian Res. 2015; 8:56. https://doi.org/10.1186/s13048-015-0186-7. [PubMed]
95. Vilming Elgaaen B, Olstad OK, Haug KB, Brusletto B, Sandvik L, Staff AC, Gautvik KM, Davidson B. Global miRNA expression analysis of serous and clear cell ovarian carcinomas identifies differentially expressed miRNAs including miR-200c-3p as a prognostic marker. BMC Cancer. 2014; 14:80. https://doi.org/10.1186/1471-2407-14-80. [PubMed]
96. Niu K, Shen W, Zhang Y, Zhao Y, Lu Y. MiR-205 promotes motility of ovarian cancer cells via targeting ZEB1. Gene. 2015; 574:330–36. https://doi.org/10.1016/j.gene.2015.08.017. [PubMed]
97. Zheng H, Zhang L, Zhao Y, Yang D, Song F, Wen Y, Hao Q, Hu Z, Zhang W, Chen K. Plasma miRNAs as diagnostic and prognostic biomarkers for ovarian cancer. PLoS One. 2013; 8:e77853. https://doi.org/10.1371/journal.pone.0077853. [PubMed]
98. Li J, Li L, Li Z, Gong G, Chen P, Liu H, Wang J, Liu Y, Wu X. The role of miR-205 in the VEGF-mediated promotion of human ovarian cancer cell invasion. Gynecol Oncol. 2015; 137:125–33. https://doi.org/10.1016/j.ygyno.2015.01.531. [PubMed]
99. Yan J, Jiang JY, Meng XN, Xiu YL, Zong ZH. MiR-23b targets cyclin G1 and suppresses ovarian cancer tumorigenesis and progression. J Exp Clin Cancer Res. 2016; 35:31. https://doi.org/10.1186/s13046-016-0307-1. [PubMed]
100. Li N, Kaur S, Greshock J, Lassus H, Zhong X, Wang Y, Leminen A, Shao Z, Hu X, Liang S, Katsaros D, Huang Q, Bützow R, et al. A combined array-based comparative genomic hybridization and functional library screening approach identifies mir-30d as an oncomir in cancer. Cancer Res. 2012; 72:154–64. https://doi.org/10.1158/0008-5472.CAN-11-2484. [PubMed]
101. LaCroix B, Gamazon ER, Lenkala D, Im HK, Geeleher P, Ziliak D, Cox NJ, Huang RS. Integrative analyses of genetic variation, epigenetic regulation, and the transcriptome to elucidate the biology of platinum sensitivity. BMC Genomics. 2014; 15:292. https://doi.org/10.1186/1471-2164-15-292. [PubMed]
102. Ye Z, Zhao L, Li J, Chen W, Li X. miR-30d Blocked Transforming Growth Factor β1-Induced Epithelial-Mesenchymal Transition by Targeting Snail in Ovarian Cancer Cells. Int J Gynecol Cancer. 2015; 25:1574–81. https://doi.org/10.1097/IGC.0000000000000546. [PubMed]
103. Creighton CJ, Fountain MD, Yu Z, Nagaraja AK, Zhu H, Khan M, Olokpa E, Zariff A, Gunaratne PH, Matzuk MM, Anderson ML. Molecular profiling uncovers a p53-associated role for microRNA-31 in inhibiting the proliferation of serous ovarian carcinomas and other cancers. Cancer Res. 2010; 70:1906–15. https://doi.org/10.1158/0008-5472.CAN-09-3875. [PubMed]
104. Samuel P, Pink RC, Caley DP, Currie JM, Brooks SA, Carter DR. Over-expression of miR-31 or loss of KCNMA1 leads to increased cisplatin resistance in ovarian cancer cells. Tumour Biol. 2016; 37:2565–73. https://doi.org/10.1007/s13277-015-4081-z. [PubMed]

105. Cao J, Cai J, Huang D, Han Q, Chen Y, Yang Q, Yang C, Kuang Y, Li D, Wang Z. miR-335 represents an independent prognostic marker in epithelial ovarian cancer. Am J Clin Pathol. 2014; 141:437–42. https://doi.org/10.1309/AJCPLYTZGB54ISZC. [PubMed]

106. Cao J, Cai J, Huang D, Han Q, Yang Q, Li T, Ding H, Wang Z. miR-335 represents an invasion suppressor gene in ovarian cancer by targeting Bcl-w. Oncol Rep. 2013; 30:701–06. https://doi.org/10.3892/or.2013.2482. [PubMed]

107. Lin Y, Xu T, Zhou S, Cui M. MicroRNA-363 inhibits ovarian cancer progression by inhibiting NOB1. Oncotarget. 2017; 8:101649–58. https://doi.org/10.18632/oncotarget.21417. [PubMed]

108. Quitadamo A, Tian L, Hall B, Shi X. An integrated network of microRNA and gene expression in ovarian cancer. BMC Bioinformatics. 2015 (Suppl 5); 16:S5. https://doi.org/10.1186/1471-2105-16-S5-S5. [PubMed]

109. Cheng Y, Ban R, Liu W, Wang H, Li S, Yue Z, Zhu G, Zhuan Y, Wang C. MiRNA-409-3p enhances cisplatin-sensitivity of ovarian cancer cells by blocking the autophagy mediated by Fip200. Oncol Res. 2018 Jan 2. https://doi.org/10.3727/096504017X15138991620238. [Epub ahead of print]. [PubMed]

110. Wang L, Mezencev R, Švajdler M, Benigno BB, McDonald JF. Ectopic over-expression of miR-429 induces mesenchymal-to-epithelial transition (MET) and increased drug sensitivity in metastasizing ovarian cancer cells. Gynecol Oncol. 2014; 134:96–103. https://doi.org/10.1016/j.ygyno.2014.04.055. [PubMed]

111. Chen J, Wang L, Matyunina LV, Hill CG, McDonald JF. Overexpression of miR-429 induces mesenchymal-to-epithelial transition (MET) in metastatic ovarian cancer cells. Gynecol Oncol. 2011; 121:200–05. https://doi.org/10.1016/j.ygyno.2010.12.339. [PubMed]

112. Yang A, Wang X, Yu C, Jin Z, Wei L, Cao J, Wang Q, Zhang M, Zhang L, Zhang L, Hao C. microRNA-494 is a potential prognostic marker and inhibits cellular proliferation, migration and invasion by targeting SIRT1 in epithelial ovarian cancer. Oncol Lett. 2017; 14:3177–84. https://doi.org/10.3892/ol.2017.6501. [PubMed]

113. Wang W, Ren F, Wu Q, Jiang D, Li H, Shi H. MicroRNA-497 suppresses angiogenesis by targeting vascular endothelial growth factor A through the PI3K/AKT and MAPK/ERK pathways in ovarian cancer. Oncol Rep. 2014; 32:2127–33. https://doi.org/10.3892/or.2014.3439. [PubMed]

114. Xu S, Fu GB, Tao Z, OuYang J, Kong F, Jiang BH, Wan X, Chen K. MiR-497 decreases cisplatin resistance in ovarian cancer cells by targeting mTOR/P70S6K1. Oncotarget. 2015; 6:26457–71. https://doi.org/10.18632/oncotarget.4762. [PubMed]

115. Lin Z, Zhao J, Wang X, Zhu X, Gong L. Overexpression of microRNA-497 suppresses cell proliferation and induces apoptosis through targeting paired box 2 in human ovarian cancer. Oncol Rep. 2016; 36:2101–07. https://doi.org/10.3892/or.2016.5012. [PubMed]

116. Boyerinas B, Park SM, Murmann AE, Gwin K, Montag AG, Zillhardt M, Hua YJ, Lengyel E, Peter ME. Let-7 modulates acquired resistance of ovarian cancer to Taxanes via IMP-1-mediated stabilization of multidrug resistance 1. Int J Cancer. 2012; 130:1787–97. https://doi.org/10.1002/ijc.26190. [PubMed]

117. Wiemer EA, Berns EM. MicroRNA Regulation of RAD51 in Serous Ovarian Cancer and Chemotherapy Response. Oxford University Press US; 2015.

118. Sun Y, Hu L, Zheng H, Bagnoli M, Guo Y, Rupaimoole R, Rodriguez-Aguayo C, Lopez-Berestein G, Ji P, Chen K, Sood AK, Mezzanzanica D, Liu J, et al. MiR-506 inhibits multiple targets in the epithelial-to-mesenchymal transition network and is associated with good prognosis in epithelial ovarian cancer. J Pathol. 2015; 235:25–36. https://doi.org/10.1002/path.4443. [PubMed]

119. Liu G, Sun Y, Ji P, Li X, Cogdell D, Yang D, Parker Kerrigan BC, Shmulevich I, Chen K, Sood AK, Xue F, Zhang W. MiR-506 suppresses proliferation and induces senescence by directly targeting the CDK4/6-FOXM1 axis in ovarian cancer. J Pathol. 2014; 233:308–18. https://doi.org/10.1002/path.4348. [PubMed]

120. Chen W, Zeng W, Li X, Xiong W, Zhang M, Huang Y, Zhou L, Jiang S. MicroRNA-509-3p increases the sensitivity of epithelial ovarian cancer cells to cisplatin-induced apoptosis. Pharmacogenomics. 2016; 17:187–97. https://doi.org/10.2217/pgs.15.166. [PubMed]

121. Pan Y, Robertson G, Pedersen L, Lim E, Hernandez-Herrera A, Rowat AC, Patil SL, Chan CK, Wen Y, Zhang X, Basu-Roy U, Mansukhani A, Chu A, et al. miR-509-3p is clinically significant and strongly attenuates cellular migration and multi-cellular spheroids in ovarian cancer. Oncotarget. 2016; 7:25930–48. https://doi.org/10.18632/oncotarget.8412. [PubMed]

122. Zhong X, Li N, Liang S, Huang Q, Coukos G, Zhang L. Identification of microRNAs regulating reprogramming factor LIN28 in embryonic stem cells and cancer cells. J Biol Chem. 2010; 285:41961–71. https://doi.org/10.1074/jbc.M110.169607. [PubMed]

123. Guo LM, Pu Y, Han Z, Liu T, Li YX, Liu M, Li X, Tang H. MicroRNA-9 inhibits ovarian cancer cell growth through regulation of NF-κB1. FEBS J. 2009; 276:5537–46. https://doi.org/10.1111/j.1742-4658.2009.07237.x. [PubMed]

124. Zhao HM, Wei W, Sun YH, Gao JH, Wang Q, Zheng JH. MicroRNA-9 promotes tumorigenesis and mediates sensitivity to cisplatin in primary epithelial ovarian cancer cells. Tumour Biol. 2015; 36:6867–73. https://doi.org/10.1007/s13277-015-3399-x. [PubMed]

125. Sun C, Li N, Yang Z, Zhou B, He Y, Weng D, Fang Y, Wu P, Chen P, Yang X, Ma D, Zhou J, Chen G. miR-9 regulation of BRCA1 and ovarian cancer sensitivity to cisplatin and PARP inhibition. J Natl Cancer Inst. 2013; 105:1750–58. https://doi.org/10.1093/jnci/djt302. [PubMed]

126. Lee H, Park CS, Deftereos G, Morihara J, Stern JE, Hawes SE, Swisher E, Kiviat NB, Feng Q. MicroRNA expression in ovarian carcinoma and its correlation with

clinicopathological features. World J Surg Oncol. 2012; 10:174. https://doi.org/10.1186/1477-7819-10-174. [PubMed]

127. Fu X, Tian J, Zhang L, Chen Y, Hao Q. Involvement of microRNA-93, a new regulator of PTEN/Akt signaling pathway, in regulation of chemotherapeutic drug cisplatin chemosensitivity in ovarian cancer cells. FEBS Lett. 2012; 586:1279–86. https://doi.org/10.1016/j.febslet.2012.03.006. [PubMed]

128. Helland Å, Anglesio MS, George J, Cowin PA, Johnstone CN, House CM, Sheppard KE, Etemadmoghadam D, Melnyk N, Rustgi AK, Phillips WA, Johnsen H, Holm R, et al, and Australian Ovarian Cancer Study Group. Deregulation of MYCN, LIN28B and LET7 in a molecular subtype of aggressive high-grade serous ovarian cancers. PLoS One. 2011; 6:e18064. https://doi.org/10.1371/journal.pone.0018064. [PubMed]

129. Wang Y, Hu X, Greshock J, Shen L, Yang X, Shao Z, Liang S, Tanyi JL, Sood AK, Zhang L. Genomic DNA copy-number alterations of the let-7 family in human cancers. PLoS One. 2012; 7:e44399. https://doi.org/10.1371/journal.pone.0044399. [PubMed]

130. Wendler A, Keller D, Albrecht C, Peluso JJ, Wehling M. Involvement of let-7/miR-98 microRNAs in the regulation of progesterone receptor membrane component 1 expression in ovarian cancer cells. Oncol Rep. 2011; 25:273–79. https://doi.org/10.3892/or_00001071. [PubMed]

131. Park SM, Shell S, Radjabi AR, Schickel R, Feig C, Boyerinas B, Dinulescu DM, Lengyel E, Peter ME. Let-7 prevents early cancer progression by suppressing expression of the embryonic gene HMGA2. Cell Cycle. 2007; 6:2585–90. https://doi.org/10.4161/cc.6.21.4845. [PubMed]

132. Guo R, Abdelmohsen K, Morin PJ, Gorospe M. Novel microRNA reporter uncovers repression of let-7 by GSK-3β. PLoS One. 2013; 8:e66330. https://doi.org/10.1371/journal.pone.0066330. [PubMed]

133. Sun H, Shao Y, Huang J, Sun S, Liu Y, Zhou P, Yang H. Prognostic value of microRNA-9 in cancers: a systematic review and meta-analysis. Oncotarget. 2016; 7:67020–32. https://doi.org/10.18632/oncotarget.11466. [PubMed]

134. Taylor DD, Gercel-Taylor C. MicroRNA signatures of tumor-derived exosomes as diagnostic biomarkers of ovarian cancer. Gynecol Oncol. 2008; 110:13–21. https://doi.org/10.1016/j.ygyno.2008.04.033. [PubMed]

135. Li LW, Xiao HQ, Ma R, Yang M, Li W, Lou G. miR-152 is involved in the proliferation and metastasis of ovarian cancer through repression of ERBB3. Int J Mol Med. 2018; 41:1529–35. https://doi.org/10.3892/ijmm.2017.3324. [PubMed]

136. Kinose Y, Sawada K, Nakamura K, Sawada I, Toda A, Nakatsuka E, Hashimoto K, Mabuchi S, Takahashi K, Kurachi H, Lengyel E, Kimura T. The hypoxia-related microRNA miR-199a-3p displays tumor suppressor functions in ovarian carcinoma. Oncotarget. 2015; 6:11342–56. https://doi.org/10.18632/oncotarget.3604. [PubMed]

137. Jiang JH, Lv QY, Yi YX, Liao J, Wang XW, Zhang W. MicroRNA-200a promotes proliferation and invasion of ovarian cancer cells by targeting PTEN. Eur Rev Med Pharmacol Sci. 2018; 22:6260–67. https://doi.org/10.26355/eurrev_201810_16033. [PubMed]

138. Xiaohong Z, Lichun F, Na X, Kejian Z, Xiaolan X, Shaosheng W. MiR-203 promotes the growth and migration of ovarian cancer cells by enhancing glycolytic pathway. Tumour Biol. 2016; 37:14989–97. https://doi.org/10.1007/s13277-016-5415-1. [PubMed]

139. Wang G, Fu Y, Liu G, Ye Y, Zhang X. miR-218 inhibits proliferation, migration, and EMT of gastric cancer cells by targeting WASF3. Oncol Res. 2017; 25:355–64. https://doi.org/10.3727/096504016X14738114257367. [PubMed]

140. Xie X, Huang Y, Chen L, Wang J. miR-221 regulates proliferation and apoptosis of ovarian cancer cells by targeting BMF. Oncol Lett. 2018; 16:6697–704. https://doi.org/10.3892/ol.2018.9446. [PubMed]

141. Yuan JM, Shi XJ, Sun P, Liu JX, Wang W, Li M, Ling FY. Downregulation of cell cycle-related proteins in ovarian cancer line and cell cycle arrest induced by microRNA. Int J Clin Exp Med. 2015; 8:18476–81. [PubMed]

142. Zhao X, Zhou Y, Chen YU, Yu F. miR-494 inhibits ovarian cancer cell proliferation and promotes apoptosis by targeting FGFR2. Oncol Lett. 2016; 11:4245–51. https://doi.org/10.3892/ol.2016.4527. [PubMed]

143. Zhao L, Wang W, Xu L, Yi T, Zhao X, Wei Y, Vermeulen L, Goel A, Zhou S, Wang X. Integrative network biology analysis identifies miR-508-3p as the determinant for the mesenchymal identity and a strong prognostic biomarker of ovarian cancer. Oncogene. 2019; 38:2305–19. https://doi.org/10.1038/s41388-018-0577-5. [PubMed]

144. Zhang S, Zhang JY, Lu LJ, Wang CH, Wang LH. MiR-630 promotes epithelial ovarian cancer proliferation and invasion via targeting KLF6. Eur Rev Med Pharmacol Sci. 2017; 21:4542–47. [PubMed]

145. Calura E, Paracchini L, Fruscio R, DiFeo A, Ravaggi A, Peronne J, Martini P, Sales G, Beltrame L, Bignotti E, Tognon G, Milani R, Clivio L, et al. A prognostic regulatory pathway in stage I epithelial ovarian cancer: new hints for the poor prognosis assessment. Ann Oncol. 2016; 27:1511–19. https://doi.org/10.1093/annonc/mdw210. [PubMed]

146. Xie J, Liu M, Li Y, Nie Y, Mi Q, Zhao S. Ovarian tumor-associated microRNA-20a decreases natural killer cell cytotoxicity by downregulating MICA/B expression. Cell Mol Immunol. 2014; 11:495–502. https://doi.org/10.1038/cmi.2014.30. [PubMed]

147. Wang S, Zhao X, Wang J, Wen Y, Zhang L, Wang D, Chen H, Chen Q, Xiang W. Upregulation of microRNA-203 is associated with advanced tumor progression and poor prognosis in epithelial ovarian cancer. Med Oncol. 2013; 30:681. https://doi.org/10.1007/s12032-013-0681-x. [PubMed]

148. Lv T, Song K, Zhang L, Li W, Chen Y, Diao Y, Yao Q, Liu P. miRNA-34a decreases ovarian cancer cell proliferation and chemoresistance by targeting HDAC1. Biochem Cell Biol.

2018; 96:663–71. https://doi.org/10.1139/bcb-2018-0031. [PubMed]

149. Hong F, Li Y, Xu Y, Zhu L. Prognostic significance of serum microRNA-221 expression in human epithelial ovarian cancer. J Int Med Res. 2013; 41:64–71. https://doi.org/10.1177/0300060513475759. [PubMed]

150. Marchini S, Cavalieri D, Fruscio R, Calura E, Garavaglia D, Fuso Nerini I, Mangioni C, Cattoretti G, Clivio L, Beltrame L, Katsaros D, Scarampi L, Menato G, et al. Association between miR-200c and the survival of patients with stage I epithelial ovarian cancer: a retrospective study of two independent tumour tissue collections. Lancet Oncol. 2011; 12:273–85. https://doi.org/10.1016/S1470-2045(11)70012-2. [PubMed]

151. Sestito R, Cianfrocca R, Rosanò L, Tocci P, Semprucci E, Di Castro V, Caprara V, Ferrandina G, Sacconi A, Blandino G, Bagnato A. miR-30a inhibits endothelin A receptor and chemoresistance in ovarian carcinoma. Oncotarget. 2016; 7:4009–23. https://doi.org/10.18632/oncotarget.6546. [PubMed]

152. Liu G, Yang D, Rupaimoole R, Pecot CV, Sun Y, Mangala LS, Li X, Ji P, Cogdell D, Hu L, Wang Y, Rodriguez-Aguayo C, Lopez-Berestein G, et al. Augmentation of response to chemotherapy by microRNA-506 through regulation of RAD51 in serous ovarian cancers. J Natl Cancer Inst. 2015; 107. https://doi.org/10.1093/jnci/djv108. [PubMed]

153. He J, Yu JJ, Xu Q, Wang L, Zheng JZ, Liu LZ, Jiang BH. Downregulation of ATG14 by EGR1-MIR152 sensitizes ovarian cancer cells to cisplatin-induced apoptosis by inhibiting cyto-protective autophagy. Autophagy. 2015; 11:373–84. https://doi.org/10.1080/15548627.2015.1009781. [PubMed]

154. Cui Y, Wu F, Tian D, Wang T, Lu T, Huang X, Zhang P, Qin L. miR-199a-3p enhances cisplatin sensitivity of ovarian cancer cells by targeting ITGB8. Oncol Rep. 2018; 39:1649–57. https://doi.org/10.3892/or.2018.6259. [PubMed]

155. Zhou Y, Wang M, Wu J, Jie Z, Chang S, Shuang T. The clinicopathological significance of miR-1307 in chemotherapy resistant epithelial ovarian cancer. J Ovarian Res. 2015; 8:23. https://doi.org/10.1186/s13048-015-0143-5. [PubMed]

156. Liu J, Zhang X, Huang Y, Zhang Q, Zhou J, Zhang X, Wang X. miR-200b and miR-200c co-contribute to the cisplatin sensitivity of ovarian cancer cells by targeting DNA methyltransferases. Oncol Lett. 2019; 17:1453–60. https://doi.org/10.3892/ol.2018.9745. [PubMed]

157. Liu Y, Han S, Li Y, Liu Y, Zhang D, Li Y, Zhang J. MicroRNA-20a contributes to cisplatin-resistance and migration of OVCAR3 ovarian cancer cell line. Oncol Lett. 2017; 14:1780–86. https://doi.org/10.3892/ol.2017.6348. [PubMed]

158. Yu X, Chen Y, Tian R, Li J, Li H, Lv T, Yao Q. miRNA-21 enhances chemoresistance to cisplatin in epithelial ovarian cancer by negatively regulating PTEN. Oncol Lett. 2017; 14:1807–10. https://doi.org/10.3892/ol.2017.6324. [PubMed]

159. Jin AH, Zhou XP, Zhou FZ. [Inhibition of microRNA-23a increases cisplatin sensitivity of ovarian cancer cells: the possible molecular mechanisms]. [Article in Chinese]. Nan Fang Yi Ke Da Xue Xue Bao. 2015; 35:125–28. [PubMed]

160. Jin AH, Wei ZL. Molecular mechanism of increased sensitivity of cisplatin to ovarian cancer by inhibition of microRNA-23a expression. Int J Clin Exp Med. 2015; 8:13329–34. [PubMed]

161. Liu J, Wu X, Liu H, Liang Y, Gao X, Cai Z, Wang W, Zhang H. Expression of microRNA-30a-5p in drug-resistant and drug-sensitive ovarian cancer cell lines. Oncol Lett. 2016; 12:2065–70. https://doi.org/10.3892/ol.2016.4831. [PubMed]

162. Xiao S, Li Y, Pan Q, Ye M, He S, Tian Q, Xue M. MiR-34c/SOX9 axis regulates the chemoresistance of ovarian cancer cell to cisplatin-based chemotherapy. J Cell Biochem. 2019; 120:2940–53. https://doi.org/10.1002/jcb.26865. [PubMed]

163. Zou J, Liu L, Wang Q, Yin F, Yang Z, Zhang W, Li L. Downregulation of miR-429 contributes to the development of drug resistance in epithelial ovarian cancer by targeting ZEB1. Am J Transl Res. 2017; 9:1357–68. [PubMed]

164. Eoh KJ, Lee SH, Kim HJ, Lee JY, Kim S, Kim SW, Kim YT, Nam EJ. MicroRNA-630 inhibitor sensitizes chemoresistant ovarian cancer to chemotherapy by enhancing apoptosis. Biochem Biophys Res Commun. 2018; 497:513–20. https://doi.org/10.1016/j.bbrc.2018.02.062. [PubMed]

165. Zhan Y, Xiang F, Wu R, Xu J, Ni Z, Jiang J, Kang X. MiRNA-149 modulates chemosensitivity of ovarian cancer A2780 cells to paclitaxel by targeting MyD88. J Ovarian Res. 2015; 8:48. https://doi.org/10.1186/s13048-015-0178-7. [PubMed]

166. Chen WT, Yang YJ, Zhang ZD, An Q, Li N, Liu W, Yang B. MiR-1307 promotes ovarian cancer cell chemoresistance by targeting the ING5 expression. J Ovarian Res. 2017; 10:1. https://doi.org/10.1186/s13048-016-0301-4. [PubMed]

167. Chen N, Chon HS, Xiong Y, Marchion DC, Judson PL, Hakam A, Gonzalez-Bosquet J, Permuth-Wey J, Wenham RM, Apte SM, Cheng JQ, Sellers TA, Lancaster JM. Human cancer cell line microRNAs associated with in vitro sensitivity to paclitaxel. Oncol Rep. 2014; 31:376–83. https://doi.org/10.3892/or.2013.2847. [PubMed]

168. Chen S, Chen X, Xiu YL, Sun KX, Zong ZH, Zhao Y. microRNA 490-3P enhances the drug-resistance of human ovarian cancer cells. J Ovarian Res. 2014; 7:84. https://doi.org/10.1186/s13048-014-0084-4. [PubMed]

Supplementary Table 1: Differential miRNAs expression between healthy tissue/benign tissue and ovarian cancer. See Supplementary Table 1

Supplementary Table 2: miRNAs related to cell proliferation/migration and invasion. See Supplementary Table 2

Supplementary Table 3: miRNAs related to overall survival. See Supplementary Table 3

Supplementary Table 4: miRNAs related to resistance to platinum agents

miRNA	Tissue*	Cell line*	miRNA expression***	Target	Resistance to Cisplatin ***	Reference Article
miR-106a	no	yes	upregulated	reduces PDCD4	increased	[4]
miR-106a	no	yes	downregulated	increases mcl-1	increased	[5]
miR-125b	no	yes	upregulated	NA	increased	[7]
miR-133a	no	yes	upregulated	reduces SUMO1 mRNA	increased	[7]
miR-130b	yes	yes	downregulated	increases CSF1	increased	[14]
miR-130b	no	yes	upregulated	reduces MDR1 and P-gp	increased	[15]
miR-141	yes	yes	upregulated	reduces KEAP	increased	[28]
miR-141	no	yes	upregulated	reduce EPHA7 and PI15	increased	[25]
miR-149	yes	yes	upregulated	decreases XIAP	decreased	[41]
miR-152	yes	yes	downregulated	increases ATG14	increased	[153]
miR-152	no	yes	upregulated	inhibits DNMT1	decreased	[102]
miR-155	no	yes	upregulated	reduces XIAP	decreased	[47]
miR-182	yes	yes	upregulated	reduces PDCD4	increased	[52]
miR-193b	no	yes	upregulated	NA	increased	[7]
miR-193b	no	yes	upregulated	inhibits MAPK	increased	[56]
miR-199a	no	yes	upregulated	reduces mTOR	decreased	[57]
miR-199a	yes	yes	upregulated	reduces CD44 and ABCG2	decreased	[58]
miR-19a-3p	no	yes	downregulated	NA	increased	[25]
miR-199a-3p	no	yes	upregulated	decreases ITGB8	decreased	[154]
miR-199a-3p	yes	yes	downregulated	increases DDR1	increased	[59]
miR-1307	yes	yes	upregulated	reduces DAPK3	increased	[155]
miR-200b	yes	yes	upregulated	decreases DNMT	decreased	[156]
miR-200c	yes	yes	upregulated	decreases DNMT	decreased	[156]
miR-215	no	yes	upregulated	NA	increased	[25]
miR-20a	no	yes	upregulated	NA	increased	[157]
miR-21	no	yes	upregulated	inhibits PDCD4	increased	[64]
miR-21	no	yes	upregulated	reduces PTEN	increased	[158]
miR-23a	no	yes	downregulated	inhibits P-gp	decreased	[159]
miR-23a	no	yes	downregulated	inhibits P-gp	decreased	[160]
miR-27a	yes	yes	upregulated	increases P-gp and MDR1	increased	[77]
miR-3a5p	no	yes	upregulated	NA	increased	[161]
miR-30d	no	yes	upregulated	decreases ABCD2	decreased	[101]
miR-31	yes	yes	upregulated	represses KCNMA1	increased	[104]
miR-34a	yes	yes	upregulated	NA	increased	[7]
miR-34a	yes	yes	upregulated	decreases HDAC1	decreased	[148]

miR-34c	yes	yes	upregulated	decreases SOX9, β-catenin, c-Myc	decreased	[162]
miR-363	yes	yes	upregulated	decreases SNAIL	decreased	[46]
miR-409-3p	yes	yes	downregulated	NA	increased	[23]
miR-429	yes	yes	downregulated	increases ZEB1	increased	[163]
miR-497	yes	yes	upregulated	mTOR/P70S6K1	decreased	[114]
miR-506	yes	yes	upregulated	reduces RAD51	decreased	[152]
miR-509-3p	no	yes	upregulated	reduces XIAP	decreased	[120]
miR-603	no	yes	downregulated	NA	decreased	[164]
miR-9	yes	yes	upregulated	reduces BRCA1	decreased	[125]
miR-9	yes	no	upregulated	NA	decreased	[124]
miR-93	yes	yes	upregulated	reduces PTEN-AKT	increased	[127]
let-7c	no	yes	downregulated	NA	increased	[133]
let-7i	no	yes	upregulated	represes PGRMC1	decreased	[134]

*It was accomplished in healthy ovarian human tissue
**It was accomplished in human cell line and human cell culture
***It was correlated the level of microRNA and resistance to cisplatin
miRNA (microRNA)

Supplementary Table 5: miRNAs related to resistance to paclitaxel

miRNA	**Tissue***	**Cell line***	**miRNA expression*****	**Target**	**Resistance to paclitaxel*****	**Reference Article**
miR-106a	yes	yes	upregulated	reduces BCL10 and caspase-7	increased	[2]
miR-130b	yes	yes	downregulated	increases CSF-1	increased	[14]
miR-130b	no	yes	upregulated	reduces MDR1 and P-gp	increased	[15]
miR-141	no	yes	downregulated	decreases ZEB1, ZEB2, SNAI2, E-cadherin and increases fibronectin and vimetin	increased	[29]
miR-145	yes	yes	downregulated	increases Sp1 and Cdk6	increased	[35]
miR-148a	yes	yes	upregulated	increases PDIA3	increased	[40]
miR-149	no	yes	downregulated	increases BCL2 and decreases BAX	increased	[165]
miR-182	yes	yes	upregulated	reduces PDCD4	increased	[52]
miR-199a	yes	yes	upregulated	reduces CD44 and ABCG2	reduced	[58]
miR-1307	no	yes	upregulated	decreases ING5	increased	[166]
miR-1307	no	yes	upregulated	reduces DAPK3	increased	[155]
miR-21	yes	yes	upregulated	reduces APAF1	increased	[60]
miR-27a	no	yes	upregulated	increases MDR1 and P-gp protein and decreases HIPK2	increased	[75]
miR-29b	no	yes	upregulated	reduces PTEN, MAPK4 and IGF1	decreased	[80]
miR-200a	no	yes	upregulated	NA	decreased	[86]
miR-200c	no	yes	downregulated	increases ZEB2 and reduces E-caderina	increased	[90]
miR-200c	no	yes	upregulated	reduces TUBB3	decreased	[92]
miR-200c	no	yes	upregulated	reduces TUBB3	decreased	[93]
miR-215	no	yes	upragulated	NA	decreased	[25]
miR-30a-5p	no	yes	upregulated	NA	increased	[161]
miR-30a-5p	no	yes	upregulated	NA	increased	[167]
miR-490-3p	no	yes	upregulated	increases MDR1/P-gp	increased	[168]
let 7i	no	yes	upregulated	represses PGRMC1	decreased	[134]

*It was accomplished in healthy ovarian human tissue

**It was accomplished in human cell line and human cell culture

***It was correlated the level of microRNA and resistance to paclitaxel

miRNA= microRNA
