# Supplementary material for: The prognostic role of microRNA in epithelial ovarian cancer: a systematic review of literature with an overall survival meta-analysis
Source: Oncotarget. 2020 Mar 24;11(12):1085–95. doi: 10.18632/oncotarget.27246 (PMC7105164; doi:10.18632/oncotarget.27246)
Supplement: Supplementary file 2 [file oncotarget-11-1085-s002.docx]

**Supplementary Table 1: Differential miRNAs expression between healthy tissue/benign tissue and ovarian cancer**

| ***miRNA*** | **Tissue*** | **Cell line **** | **miRNA expression ovarian cancer vs healthy/benign***** | **Reference Article** |
| --- | --- | --- | --- | --- |
| *miR-106a* | no | yes | upregulated | [1] |
| *miR-106a* | yes | yes | downregulated | [2] |
| *miR-106a* | yes | yes | upregulated | [3] |
| *miR-106a* | no | yes | upregulated | [4] |
| *miR-106a* | no | yes | downregulated | [5] |
| *miR-125b* | yes | yes | upregulated | [6] |
| *miR-125b* | no | yes | upregulated | [7] |
| *miR-125b* | yes | yes | upregulated | [8] |
| *miR-125b* | no | yes | upregulated | [9] |
| *miR-125b* | yes | yes | upregulated | [10] |
| *miR-125b* | no | yes | upregulated | [11] |
| *miR-125b* | no | yes | upregulated | [12] |
| *miR-126* | yes | no | upregulated | [13] |
| *miR-130b* | yes | yes | downregulated | [14] |
| *miR-130b* | no | yes | upregulated | [15] |
| *miR-130b* | yes | yes | upregulated | [16] |
| *miR-133a* | no | yes | upregulated | [7] |
| *miR-133a* | yes | yes | upregulated | [17] |
| *miR-133a* | yes | yes | downregulated | [18] |
| *miR-137* | yes | yes | upregulated | [19] |
| *miR-137* | yes | yes | downregulated | [20] |
| *miR-137* | no | yes | upregulated | [21] |
| *miR-139-5p* | yes | yes | downregulated | [22] |
| *miR-139-5p* | yes | yes | downregulated | [23] |
| *miR-1307* | yes | yes | upregulated | [24] |
| *miR-141* | no | yes | upregulated | [25] |
| *miR-141* | no | yes | upregulated | [1] |
| *miR-141* | yes | yes | upregulated | [26] |
| *miR-141* | yes | yes | upregulated | [27] |
| *miR-141* | yes | yes | upregulated | [28] |
| *miR-141* | no | yes | downregulated | [29] |
| *miR-145* | yes | yes | downregulated | [26] |
| *miR-145* | no | yes | upregulated | [30] |
| *miR-145* | yes | yes | upregulated | [31] |
| *miR-145* | yes | yes | upregulated | [32] |
| *miR-145* | no | yes | upregulated | [33] |
| *miR-145* | yes | yes | upregulated | [34] |
| *miR-145* | yes | yes | downregulated | [35] |
| *miR-145* | yes | yes | upregulated | [36] |
| *miR-148a* | yes | yes | downregulated | [37] |
| *miR-148a* | yes | yes | downregulated | [38] |
| *miR-148a* | yes | yes | upregulated | [39] |
| *miR-148a* | yes | yes | upregulated | [40] |
| *miR-149* | yes | yes | downregulated | [41] |
| *miR-152* | yes | yes | downregulated | [37] |
| *miR-152* | yes | yes | downregulated | [42] |
| *miR-152* | no | yes | upregulated | [43] |
| *miR-152* | no | yes | upregulated | [44] |
| *miR-155* | no | yes | upregulated | [45] |
| *miR-155* | no | yes | upregulated | [46] |
| *miR-155* | no | yes | upregulated | [47] |
| *miR-155* | yes | no | downregulated | [48] |
| *miR-182* | no | yes | upregulated | [49] |
| *miR-182* | yes | no | upregulated | [50] |
| *miR-182* | yes | yes | upregulated | [51] |
| *miR-182* | yes | yes | upregulated | [52] |
| *miR-182* | yes | yes | upregulated | [53] |
| *miR-182* | yes | no | upregulated | [54] |
| *miR-193b* | no | yes | upregulated | [7] |
| *miR-193b* | yes | yes | downregulated | [55] |
| *miR-193b* | no | yes | upregulated | [56] |
| *miR-199a* | yes | yes | upregulated | [12] |
| *miR-199a* | no | yes | upregulated | [57] |
| *miR-199a* | yes | yes | upregulated | [58] |
| *miR-199a* | no | yes | downregulated | [11] |
| *miR-199a* | yes | yes | downregulated | [26] |
| *miR-199a-3p* | yes | yes | downregulated | [59] |
| *miR-21* | yes | no | upregulated | [48] |
| *miR-21* | yes | yes | upregulated | [60] |
| *miR-21* | yes | no | upregulated | [61] |
| *miR-21* | yes | no | upregulated | [62] |
| *miR-21* | yes | yes | downregulated | [63] |
| *miR-21* | no | yes | upregulated | [64] |
| *miR-21* | no | yes | upregulated | [65] |
| *miR-22* | no | yes | upregulated | [66] |
| *miR-22* | yes | yes | upregulated | [67] |
| *miR-22* | yes | no | downregulated | [68] |
| *miR-22* | no | yes | upregulated | [69] |
| *miR-25* | yes | yes | upregulated | [51] |
| *miR-25* | yes | no | upregulated | [70] |
| *miR-25* | yes | yes | upregulated | [71] |
| *miR-25* | yes | yes | downregulated | [72] |
| *miR-23a* | yes | no | upregulated | [73] |
| *miR-23b* | yes | no | downregulated | [73] |
| *miR-23b* | yes | yes | downregulated | [74] |
| *miR-27a* | no | yes | upregulated | [75] |
| *miR-27a* | no | yes | upregulated | [76] |
| *miR-27a* | yes | no | upregulated | [77] |
| *miR-29b* | yes | yes | downregulated | [78] |
| *miR-29b* | yes | yes | upregulated | [79] |
| *miR-29b* | no | yes | upregulated | [80] |
| *miR-200a* | yes | yes | upregulated | [27] |
| *miR-200a* | yes | yes | upregulated | [28] |
| *miR-200a* | yes | no | upregulated | [81] |
| *miR-200a* | yes | no | upregulated | [82] |
| *miR-200a* | yes | no | upregulated | [83] |
| *miR-200a* | no | yes | upregulated | [41] |
| *miR-200a* | yes | yes | upregulated | [84] |
| *miR-200a* | yes | no | downregulated | [85] |
| *miR-200a* | no | yes | upregulated | [86] |
| *miR-200a* | yes | no | upregulated | [74] |
| *miR-200b* | yes | no | upregulated | [81] |
| *miR-200b* | yes | no | upregulated | [82] |
| *miR-200b* | yes | no | upregulated | [83] |
| *miR-200b* | no | yes | upregulated | [87] |
| *miR-200b* | yes | no | upregulated | [88] |
| *miR-200c* | sim | no | upregulated | [81] |
| *miR-200c* | yes | no | upregulated | [82] |
| *miR-200c* | yes | no | upregulated | [83] |
| *miR-200c* | yes | no | upregulated | [89] |
| *miR-200c* | no | yes | downregulated | [90] |
| *miR-200c* | yes | yes | upregulated | [91] |
| *miR-200c* | no | yes | upregulated | [92] |
| *miR-200c* | no | yes | upregulated | [93] |
| *miR-200c* | yes | yes | upregulated | [94] |
| *miR-200c* | no | yes | upregulated | [1] |
| *miR-200c* | yes | yes | upregulated | [26] |
| *miR-200c-3p* | no | yes | upregulated | [95] |
| *miR-200c-3p* | yes | no | upregulated | [76] |
| *miR-200c-3p* | yes | no | upregulated | [25] |
| *miR-200c-3p* | no | yes | upregulated | [1] |
| *miR-205* | yes | yes | upregulated | [96] |
| *miR-205* | yes | no | upregulated | [97] |
| *miR-205* | no | yes | upregulated | [98] |
| *miR-221* | yes | no | upregulated | [99] |
| *miR-30d* | yes | yes | upregulated | [100] |
| *miR-30d* | no | yes | upregulated | [101] |
| *miR-30d* | no | yes | upregulated | [102] |
| *miR-31* | yes | yes | upregulated | [103] |
| *miR-31* | no | yes | upregulated | [104] |
| *miR-31* | no | yes | upregulated | [105] |
| *miR-335* | no | yes | upregulated | [25] |
| *miR-335* | yes | no | downregulated | [106] |
| *miR-335* | yes | yes | upregulated | [107] |
| *miR-363*  *miR-373* | yes  yes | yes  no | downregulated  upregulated | [108]  [82] |
| *miR-375* | yes | yes | upregulated | [24] |
| *miR-375* | no | yes | upregulated | [108] |
| *miR-409-3p* | yes | no | downregulated | [109] |
| *miR-409-3p* | no | yes | downregulated | [1] |
| *miR-429* | yes | no | upregulated | [76] |
| *miR-429* | yes | yes | upregulated | [51] |
| *miR-429* | no | yes | upregulated | [110] |
| *miR-429* | no | yes | upregulated | [111] |
| *miR-429* | yes | yes | upregulated | [51] |
| *miR-494* | no | yes | downregulated | [1] |
| *miR-494* | yes | yes | downregulated | [112] |
| *miR-497* | yes | yes | downregulated | [25] |
| *miR-497* | yes | yes | upregulated | [113] |
| *miR-497* | yes | yes | downregulated | [114] |
| *miR-497* | yes | yes | upregulated | [115] |
| *miR-506* | yes | yes | upregulated | [116] |
| *miR-506* | yes | no | upregulated | [117] |
| *miR-506* | yes | yes | upregulated | [118] |
| *miR-506* | no | yes | downregulated | [119] |
| *miR-509-3p* | no | yes | upregulated | [120] |
| *miR-509-3p* | yes | yes | upregulated | [121] |
| *miR-509-3p* | yes | no | upregulated | [76] |
| *miR-9* | no | yes | upregulated | [122] |
| *miR-9* | yes | yes | upregulated | [123] |
| *miR-9* | yes | no | upregulated | [124] |
| *miR-9* | yes | yes | upregulated | [125] |
| *miR-9* | yes | no | upregulated | [126] |
| *miR-93* | yes | yes | downregulated | [51] |
| *miR-93* | yes | no | upregulated | [67] |
| *miR-93* | yes | yes | upregulated | [127] |
| *miR-93* | yes | yes | downregulated | [51] |
| *let 7 family* | no | yes | upregulated | [122] |
| *let 7 family* | yes | yes | downregulated | [128] |
| *let 7 b* | yes | yes | upregulated | [129] |
| *let 7 family* | no | yes | downregulated | [130] |
| *let 7d* | yes | yes | downregulated | [117] |
| *let 7 family* | yes | yes | upregulated | [131] |
| *let 7 family* | no | yes | upregulated | [132] |
| *let 7 a* | yes | no | downregulated | [92] |
| *let 7c* | no | yes | downregulated | [133] |
| *let 7b* | yes | yes | downregulated | [74] |
| *let 7f* | yes | no | downregulated | [94] |
| *let 7i* | no | yes | upregulated | [134] |

- ***It was accomplished in healthy ovarian human tissue**
- ****It was accomplished in human cell line**
- *****It was compared the level of microRNA among ovarian tumor tissue/plasma/cell line versus healthy or benign tissue/plasma/cell line**
- **miRNA (microRNA)**
