# Supplementary material for: The prognostic role of microRNA in epithelial ovarian cancer: a systematic review of literature with an overall survival meta-analysis
Source: Oncotarget. 2020 Mar 24;11(12):1085–95. doi: 10.18632/oncotarget.27246 (PMC7105164; doi:10.18632/oncotarget.27246)
Supplement: Supplementary file 3 [file oncotarget-11-1085-s003.docx]

**Supplementary Table 2: miRNAs related to cell proliferation/migration and invasion**

| ***miRNA*** | **Tissue*** | **Cell line*** | **miRNA Expression***** | **Target** | **Action***** | **Reference Article** |
| --- | --- | --- | --- | --- | --- | --- |
| *miR-106a* | 33 HGSOC and 6 benign tissue | yes | upregulated | reduces BCL10 e caspase-7 | increases cell proliferation | [2] |
| *miR-106a* | 15 EOC tissue and adjacent normal tissue | yes | upregulated | increases PTEN | increases cell proliferation and invasion | [3] |
| *miR-125b* | 55 EOC tissue and adjacent normal tissue | yes | upregulated | reduces SET | inhibit EMT, migration and invasion | [6] |
| *miR-125b* | 20 EOC X 7 benign tissue | yes | upregulated | reduces EIF4, EBP1 | inhibits invasion and migration | [8] |
| *miR-125b* | 28 EOC X 11 normal tissue | yes | upregulated | reduces BCL2 | inhibits cell proliferation and invasion | [9] |
| *miR-125b* | 20 HGSOC and 10 normal tissue | yes | upregulated | reduces BLC3 | inhibits cell proliferation | [10] |
| *miR-125b* | 33 HGSOC and 7 normal tissue | yes | upregulated | inhibits HIF-1a/VEGF | inhibits angiogenesis | [12] |
| *miR-130b* | 100 EOC and 20 normal tissue | yes | upregulated | reduces RUNX3 | inhibits migration and invasion | [16] |
| *miR-133a* | 16 EOC and 8 normal tissues | yes | upregulated | inhibits IGF1R-3'UTR | inhibits cell proliferation | [17] |
| *miR-133a* | 70 EOC and 26 normal tissues | yes | upregulated | NA | reduces cell growth and induces apoptosis | [18] |
| *miR-137* | 30 EOC and 30 normal adjacent tissues | yes | upregulated | reduces MMP-2 and MMP-9 | inhibits cell growth, EMT and invasion | [19] |
| *miR-137* | 50 EOC and 50 normal adjacent tissue | yes | downregulated | increases Snail | increases EMT and cell growth | [20] |
| *miR-137* | 20 EOC and 10 normal tissues | yes | upregulated | reduces AEG-1 | inhibits EMT and cell growth | [21] |
| *miR-145* | 50 EOC and 50 adjacent normal tissues | yes | upregulated | Inhibits c-myc | inhibits cell proliferation | [31] |
| *miR-145* | 38 EOC and 6 normal tissue | yes | upregulated | reduces TRIM2 | inhibits cell proliferation | [32] |
| *miR-145* | 3 normal tissue, 12 EOC and 12 serum EOC | yes | upregulated | reduces P70S6K1 and MUC1 | inhibits cell proliferation and invasion | [34] |
| *miR-145* | 76 HGSOC | yes | upregulated | inhibits MTDH | inhibits cell growth and invasion | [33] |
| *miR-148a* | 78 EOC and 17 normal tissue | yes | upregulated | NA | inhibits cell proliferation | [37] |
| *miR-148a* | 20 EOC and 20 adjacent normal tissue | yes | upregulated | inhibits S1PR1 | inhibits cell proliferation | [39] |
| *miR-148a* | 102 EOC plasma and 70 normal tissues and plasma | yes | downregulated | NA | increases cell proliferation, migration and invasion | [38] |
| *miR-149* | 58 EOC and adjacent tissue | yes | upregulated | Blocks TGFβ1, reduces SNAIL | decreases cell proliferation | [41] |
| *miR-152* | no tissue | yes | upregulated | NA | reduces cell proliferation, migration and invasion | [135] |
| *miR-152* | 78 EOC and 17 normal tissues | yes | upregulated | NA | inhibits cell proliferation | [37] |
| *miR-182* | 13 EOC and 2 normal tissue | yes | upregulated | reduces PDCD4 | increases cell growth and migration | [52] |
| *miR-182* | 56 HGSOC and 21 normal tissue | yes | upregulated | increases HMGA2, reduces MTSS1 and *BRCA1* | increases invasion and cell proliferation | [53] |
| *miR-193b* | 7 HGSOC and 7 adjacent normal tissue | yes | downregulated | increases uPA | increases proliferation and invasion | [55] |
| *miR-199a* | 33 EOC and 7 normal tissue | yes | upregulated | inhibits P70S6K1 and VEGF, reduces HER2 and HER3 | inhibits angiogenesis and cell proliferation | [12] |
| *miR-199a* | 9 EOC | yes | upregulated | reduces CD44 and ABCG2 | inhibits cell proliferation, invasion and metastasis | [58] |
| *miR-199a-3p* | 115 EOC and adjacent normal tissue | yes | downregulated | increases DDR1 and decreases c-met | increases tumor aggressiveness | [112] |
| *miR-199a-3p* | no tissue | yes | downregulated | NA | increases cell proliferation, invasion and migration | [136] |
| *miR-22* | 31 EOC plasma and 23 benign tumors plasma and 8 normal plasma | yes | upregulated | reduces Ezrin | inhibits metastasis | [67] |
| *miR-23b* | 116 EOC and 5 normal tissue | yes | downregulated | reduces RUNX2 | increases cell proliferation | [74] |
| *miR-23b* | no tissue | yes | upregulated | increases RUNX2 | inhibits cell proliferation invasion and migration | [99] |
| *miR-25* | 18 EOC vs adjacent normal tissue | yes | upregulated | inhibits LATS2 | increases cell proliferation and motility | [71] |
| *miR-25* | 6 EOC vs 6 adjacent normal tissue | yes | downregulated | increses BIM | Inhibit cell proliferation and induces apoptosis | [72] |
| *miR-29b* | 30 EOC and 30 normal adjacent tissue | yes | upregulated | reduces AKT2 and AKT3 | inhibits cell progression | [79] |
| *miR-200a* | 57 EOC and adjacent tissue | yes | upregulated | reduces PTEN | increases invasion and migration | [137] |
| *miR-200c* | 48 EOC and 30 normal tissue | yes | upregulated | reduces ZEB2 | reduces invasion and migration | [91] |
| *miR-200c-3p* | 54 EOC and 9 normal tissue | no | upregulated | reduces ZEB1 and ZEB2 | increases metastasis | [25] |
| *miR-203* | no tissue | yes | upregulated | decreases BIRC5 | increases cell growth and migration | [138] |
| *miR-205* | 110 EOC and 20 normal tissue | yes | upregulated | reduces ZEB1 | increases cell motiliy and aggressiveness | [96] |
| *miR-205* | 360 plasma EOC and 200 normal tissue and plasma | yes | upregulated | reduces ZEB1 and ZEB2 | increases migration and invasion | [97] |
| *miR-215* | 48 EOC and adjacent normal tissue | yes | upregulated | decreases RUNX2 | decreases proliferation and migration | [139] |
| *miR-221* | 360 plasma EOC and 200 normal tissue and plasma | yes | downregulated | NA | decreases cell proliferation | [140] |
| *miR-30d* | 330 EOC | yes | upregulated | Increases CASP3 | increases cell proliferation | [100] |
| *miR-34a* | no tissue | yes | upregulated | increases BMF | decreases cell proliferation | [100] |
|  |  |  |  |  |  |  |
| *miR-34c* | no tissue | yes | upregulated | decreases AXL | decreases proliferation, migration and invasion | [141] |
| *miR-363* | 50 OC and adjacent normal tissue | yes | upregulated | decreases CDK6 and CDC25A | decreases migration and invasion | [107] |
| *miR-429* | 180 EOC and 66 normal tissue | yes | upregulated | decreases ZEB1, ZEB2 | decreases cell proliferation | [51] |
| *miR-494* | 25 OC and adjacent normal tissue | yes | upregulated | decreases NOB1 | decreases cell proliferation | [142] |
| *miR-494* | 96 OC and adjacent normal tissue | yes | upregulated | decreases FGFR2 | decreases cell proliferation | [112] |
| *miR-497* | 26 EOC and 26 adjacent normal tissue | yes | upregulated | increases PAX2 | inhibits cell proliferation and promotes apoptosis | [115] |
| *miR-497* | 30 EOC and 30 normal tissue | yes | downregulated | increases SMURF1 | increases invasion, migration and metastasis | [25] |
| *miR-497* | 96 EOC and 30 normal tissue | yes | upregulated | reduces VEGFRA and SMURF1 | inhibits angiogenesis and cell proliferation, inhibits EMT | [115] |
| *miR-506* | 468 EOC | yes | upregulated | reduces RAD51 | promotes cell death and decreases cell proliferation | [117] |
| *miR-506* | 240 EOC | yes | upregulated | reduces SNAI2 | inhibits cell proliferation, EMT and migration | [118] |
| *miR-509-3p* | 157 HGSOC | yes | upregulated | reduces YAP1 | reduces migration and cell invasion | [121] |
| *miR-508-3p* | 999 EOC | yes | upregulated | increases SIRT1 | induces EMT | [143] |
| *miR-603* | 30 EOC and normal tissue | yes | upregulated | NA | increases cell migration and proliferation | [144] |
| *miR-9* | 4 EOC and 4 adjacent normal tissue | yes | upregulated | reduces NF-κβ1 | reduces cell growth and proliferation | [123] |
| *Let 7b* | 110 EOC from TGCA | yes | upregulated | reduces HMGA2, IL6, CCND1 and LIN28B | reduces cell proliferation | [129] |

- ***It was accomplished in healthy ovarian human tissue**
- ****It was accomplished in human cell line**
- **miRNA (microRNA)**
- *****It was correlated the level of microRNA and cell proliferation, invasion and migration**
- **HGSOC= high grade serous ovarian cancer; EOC= epithelial ovarian cancer; TGCA= The Cancer Genome Atlas open access database**
