# Supplementary material for: The prognostic role of microRNA in epithelial ovarian cancer: a systematic review of literature with an overall survival meta-analysis
Source: Oncotarget. 2020 Mar 24;11(12):1085–95. doi: 10.18632/oncotarget.27246 (PMC7105164; doi:10.18632/oncotarget.27246)
Supplement: Supplementary file 4 [file oncotarget-11-1085-s004.docx]

**Supplementary Table 3: miRNAs related to overall survival**

| ***miRNA*** | **Tissue*** | **Cell line**** | **miRNA expression***** | **Target** | **OS/PFS***** | **Article reference** |
| --- | --- | --- | --- | --- | --- | --- |
| *miR-106a* | 33 serous EOC and 6 serous benign tumors | yes | downregulated | reduces BCL10 and caspase-7 | decreases OS | [1] |
| *miR-139-5p* | 46 EOC and 46 normal tissue | yes | downregulated | increases ROCK2 | decreases OS | [22] |
| *miR-141* | no tissue | yes | upregulated | NA | decreases OS | [77] |
| *miR-141* | 203 EOC | no | upregulated | NA | decreases OS | [145] |
| *miR-145* | 5 high grade serous EOC and 5 adjacent fallopian tube | yes | downregulated | increases MTDH | decreases OS | [33] |
| *miR-145* | 74 high grade serous EOC, 10 benign tumors tissues | yes | downregulated | increases HMGA2 | decreases OS | [36] |
| *miR-148a* | 102 EOC plasma and 70 normal plasma | yes | downregulated | increases S1PR1/G | decreases OS | [38] |
| *miR-149* | 58 EOC and 58 normal tissue | yes | upregulated | decreases XIAP | increases | [41] |
| *miR-182* | 47 EOC and 26 normal tissue | no | downregulated | NA | OS | [54] |
| *miR-20a* | 34 EOC and 20 normal tissue | yes | upregulated | decreases MICA/B | decreases OS | [146] |
| *miR-21* | 43 EOC and 43 adjacent tissue | NA | upregulated | increases HE4 protein | decreases OS | [61] |
| *miR-21* | 94 EOC and 40 normal serum | no | upregulated | NA | decreases OS | [62] |
| *miR-22* | 109 EOC and adjacent normal tissue | no | downregulated | increases ERα | decreases OS | [68] |
| *miR-23a* | 50 EOC and normal tissue | no | upregulated | NA | decreases OS | [73] |
| *miR-23b* | 50 EOC and normal tissue | no | downregulated | NA | decreases OS | [73] |
| *miR-23b* | 116 OC and 5 normal tissue | no | downregulated | increases RUNX2 | decreases OS | [74] |
| *miR-25* | 86 EOC and normal adjacent tissue | no | upregulated | NA | decreases OS | [70] |
| *miR-27a* | 57 endometrioid and serous EOC resistant vs sensitive to CDDP | no | upregulated | Increases P-gp and MDR1 | decreases OS | [77] |
| *miR-29b* | 160 EOC and 30 normal tissue | yes | downregulated | increases MAPK10, ATG9A, MCL1 | decreases OS | [78] |
| *miR-200a* | 100 EOC and 50 normal tissue/benign disease | no | upregulated | NA | decreases OS | [81] |
| *miR-200a* | 70 EOC and 70 normal tissue | no | upregulated | decreases ZEB1, ZEB2 and TGFβ2 repressing E-cadherin | decreases PFS | [24] |
| *miR-200a* | 55 EOC | yes | upregulated | NA | increases OS | [25] |
| *miR-200b* | 100 EOC and 50 normal tissue/benign disease | no | upregulated | NA | decreases OS | [23] |
| *miR-200b* | 163 EOC exosomes and 20 benign tumors | no | upregulated | decreases ZEB, ZEB2 | decreases OS | [26] |
| *miR-200b* | 51 EOC, 25 normal and 25 benign tumors serum | no | upregulated | NA | decreases PFS | [27] |
| *miR-200b* | 144 EOC | no | upregulated | NA | decreases OS | [37] |
| *miR-200c* | 144 EOC | no | upregulated | NA | decreases OS | [37] |
| *miR-200c* | 100 EOC and 50 normal tissues/benign disease | no | upregulated | NA | decreases OS | [74] |
| *miR-200c* | 163 EOC exosomes and 20 benign tumors | no | upregulated | decreases ZEB, ZEB2 | decreased OS | [82] |
| *miR-200c* | 70 EOC and 70 normal tissue | no | upregulated | decreases ZEB1, ZEB2 and TGFβ2 repressing E-cadherin | decreases PFS | [24] |
| *miR-200c* | 74 EOC, 19 borderline OC and 50 normal tissue | no | upregulated | NA | increases OS | [89] |
| *miR-200c-3p* | cohort study with 894 patients (3 cohorts) | no | upregulated | decreases ZEB1, ZEB2 | increases OS | [76] |
| *miR-200c-3p* | 35 high grade serous carcinoma, 19 clear cell carcinoma and 9 normal tissue | no | upregulated | reduces ZEB1 and ZEB2 and increases E-cadherin | decreases OS, PFS | [25] |
| *miR-203* | 156 EOC and normal adjacent tissue | no | upregulated | NA | decreases OS | [147] |
| *miR-221* | 63 EOC | yes | upregulated | NA | decreases OS | [148] |
| *miR-221* | 96 EOC and 35 normal adjacent tissue | yes | upregulated | NA | decreases OS | [149] |
| *miR-30d* | 330 EOC | yes | upregulated | CASP3 | decreases OS | [55] |
| *miR-30d* | 36 EOC | yes | upregulated | NA | decreases OS | [102] |
| *miR-30d* | 144 EOC | no | downregulated | NA | decreases OS | [150] |
| *miR-30d* | 109 EOC, 22 normal tissue,17 benign, 23 borderline tumors | no | downregulated | NA | decreases OS | [128] |
| *miR-30a* | 109 EOC, 22 normal tissue,17 benign, 23 borderline tumors | yes | upregulated | NA | decreases OS | [126] |
| *miR-30a* | 144 EOC | no | downregulated | NA | decreases OS | [150] |
| *miR-30a* | 112 EOC | yes | downregulated | NA | decreased OS | [151] |
| *miR-335* | 55 EOC (38 serous, 9 mucinous, 4 clear cells, 3 endometrioid and 1 undifferentiated OC) and 17 normal tissue | no | downregulated | increases Bcl-2 | decreases PFS | [105] |
| *miR-363*  *miR-373* | 50 EOC and 50 adjacent normal tissue  163 EOC exossomes and 20 benign tumors | yes  no | upregulated  upregulated | decreases NOB1  NA | decreases OS  decreases OS | [107]  [82] |
| *miR-429* | cohort study with 894 patients (3 cohorts) | no | upregulated | decreases ZEB1, ZEB2 | increases OS | [76] |
| *miR-429* | 180 EOC and 66 normal tissue | yes | upregulated | decreases ZEB1, ZEB2 | decreases OS | [51] |
| *miR-497* | 19 high grade serous cistoadenocarcinoma, 11 serous low grade OC and 30 normal tissue | yes | downregulated | increases SMURF1 | decreases OS | [25] |
| *miR-506* | 468 stage II to IV EOC cases were downloaded from TCGA | yes | upregulated | decreases RAD51 | increases OS, PFS | [152] |
| *miR-506* | 240 EOC | yes | upregulated | reduces SNAI2 vimetin and increases E-cadherin | increases OS, PFS | [118] |
| *miR-509-3p* | 157 high grade serous OC | yes | upregulated | decreases YAP1 | increases OS, PFS | [121] |
| *miR-509-3p* | cohort study with 894 patients (3 cohorts) | no | downregulated | NA | increases OS | [76] |
| *miR-9* | 58 EOC resistant and sensitive to CDDP | yes | upregulated | inhibits *BRCA1* | increases PFS | [125] |
| *let-7a* | 178 EOC with response to CDDP + paclitaxel (75) and (103) with no response | no | downregulated | NA | increases OS | [92] |
| *let-7d* | 20 EOC after surgery and after recurrence | yes | downregulated | increases IMP-1 | decreases PFS | [116] |

- ***It was accomplished in healthy ovarian human tissue**
- ****It was accomplished in human cell line and human cell culture**
- ***** It was correlated the level of microRNA to overall survival and progression-free survival**
- **miRNA= microRNA; OS= overall survival; PFS= progression free survival; EOC= epithelial ovarian cancer; OC= ovarian cancer; TGCA= The Cancer Genome Atlas open access database; CDDP= cisplatin; ERα= oestrogen receptor α; NA= not available; P-gp= P glycoprotein**
